# COPII mitigates ER stress by promoting formation of ER whorls

**DOI:** 10.1038/s41422-020-00416-2

**Published:** 2020-09-28

**Authors:** Fang Xu, Wanqing Du, Qin Zou, Yuting Wang, Xin Zhang, Xudong Xing, Ying Li, Dachuan Zhang, Huimin Wang, Wenhao Zhang, Xinyao Hu, Xin Liu, Xiaoling Liu, Shaojin Zhang, Jinqiang Yu, Jianhuo Fang, Fajin Li, Ying Zhou, Tieqiang Yue, Na Mi, Haiteng Deng, Peng Zou, Xiaowei Chen, Xuerui Yang, Li Yu

**Affiliations:** 1 The State Key Laboratory of Membrane Biology, Tsinghua University-Peking University Joint Centre for Life Sciences, Beijing Frontier Research Center for Biological Structure, School of Life Sciences, Tsinghua University, Beijing, 100084 China; 2 MOE Key Laboratory of Bioinformatics, Center for Synthetic & Systems Biology, School of Life Sciences, Tsinghua University, Beijing, 100084 China; 3 Peking-Tsinghua Center for Life Sciences, Peking University, Beijing, 100871 China; 4 National Laboratory of Biomacromolecules, CAS Center for Excellence in Biomacromolecules, Institute of Biophysics, Chinese Academy of Sciences, Beijing, 100101 China; 5 College of Chemistry and Molecular Engineering, Synthetic and Functional Biomolecules Center, Beijing National Laboratory for Molecular Sciences, Key Laboratory of Bioorganic Chemistry and Molecular Engineering of Ministry of Education, PKU-IDG/McGovern Institute for Brain Research, Peking-Tsinghua Center for Life Sciences, Peking University, Beijing, 100871 China; 6 Xinjiang Medical University, Urumqi, Xinjiang 830011 China; 7 Chinese Institute for Brain Research (CIBR), Beijing, 102206 China; 8 State Key Laboratory of Membrane Biology, Peking University, Beijing, 100871 China; 9 Institute of Molecular Medicine, Peking University, Beijing, 100871 China

**Keywords:** Endoplasmic reticulum, Collective cell migration

## Abstract

Cells mitigate ER stress through the unfolded protein response (UPR). Here, we report formation of ER whorls as an effector mechanism of the ER stress response. We found that strong ER stress induces formation of ER whorls, which contain ER-resident proteins such as the Sec61 complex and PKR-like ER kinase (PERK). ER whorl formation is dependent on PERK kinase activity and is mediated by COPII machinery, which facilitates ER membrane budding to form tubular-vesicular ER whorl precursors. ER whorl precursors then go through Sec22b-mediated fusion to form ER whorls. We further show that ER whorls contribute to ER stress-induced translational inhibition by possibly modulating PERK activity and by sequestering translocons in a ribosome-free environment. We propose that formation of ER whorls reflects a new type of ER stress response that controls inhibition of protein translation.

## Introduction

Secreted proteins and membrane proteins destined for various cellular compartments are selectively segregated from ER-resident proteins and sorted into ER-derived secretory vesicles.^1^ Cargo sorting and vesicle formation are mediated by coat protein complex II (COPII).^2^ COPII-mediated sorting is highly specific for ER-exported cargo; for example, the Sec61 complex, an ER-resident protein translocon, is not sorted into COPII vesicles.^3^ COPII-mediated sorting can also be flexible, as specific cargos can be sorted into COPII vesicles in a highly regulated manner in response to changes in the environment.^4^

Excessive accumulation of unfolded proteins in the ER induces ER stress.^5^ As a consequence, a collection of signaling pathways, together named the unfolded protein response (UPR), are activated, which mitigates the ER stress by expanding the ER, increasing the ER folding capacity and transiently shutting down protein translation.^6,7^ Among these effector mechanisms of UPR, the least understood one is ER remodeling.

Three branches of the UPR have been discovered. Among them, the PERK (double-stranded RNA-activated protein kinase (PKR)-like ER kinase) branch regulates protein translation.^8^ Upon ER stress, PERK is oligomerized and activated; the activated PERK then directly phosphorylates and inactivates the translational initiation factor eIF2α, thus leading to inhibition of translation.^8,9^ The mechanism of PERK activation is not yet fully understood. PERK activation inhibits general protein translation while promoting the translation of a selected set of proteins, including transcription factor ATF4, which further reinforces the UPR.

Multilayered concentric ER whorls have been found in various settings including Herpes Simplex Virus-infected cells.^10^ Overexpression of GFP-tagged ER-resident proteins has been shown to induce formation of Organized Smooth ER (OSER) through GFP-mediated low-affinity interactions, causing the ER membrane to zipper up into highly compacted whorls.^11^ In yeast, ER stress has been shown to induce formation of ER whorls,^12^ and the microautophagy of ER whorls, which is mediated by Nem1-Spo7 phosphatase complex and the ESCRT machinery,^13^ has been proposed as a mechanism to counter-balance ER stress-induced ER expansion.^14^ So far, the mechanism and function of ER whorl formation in mammalian cells are largely unknown.

In this study, we report formation of ER whorls through modulation of the secretory pathway as an effector mechanism of UPR. We found that prolonged ER stress induces formation of ER whorls, and this process is reversible upon removal of the ER stressor. We further demonstrated that ER whorl formation depends on PERK activation, which triggers formation of ER whorl precursors containing proteins that are usually retained in the ER. The formation of ER whorl precursors is initiated by recruitment of Sar1 and is dependent on the COPII machinery. Functionally, we demonstrated that PERK is sorted into ER whorls during ER stress, and prolonged ER stress-induced PERK activation and translational inhibition are dependent on formation of ER whorl precursors. Moreover, we found that the majority of translocons are sequestered into ER whorls during prolonged ER stress, which separates translocons from ribosomes. Based on these data, we propose that formation of ER whorls is a new type of ER stress response, which mitigates ER stress by activating PERK and modulating the import of nascent proteins into the ER.

## Results

### Formation of ER whorls in mammalian cells during ER stress

**Fig. 1:**
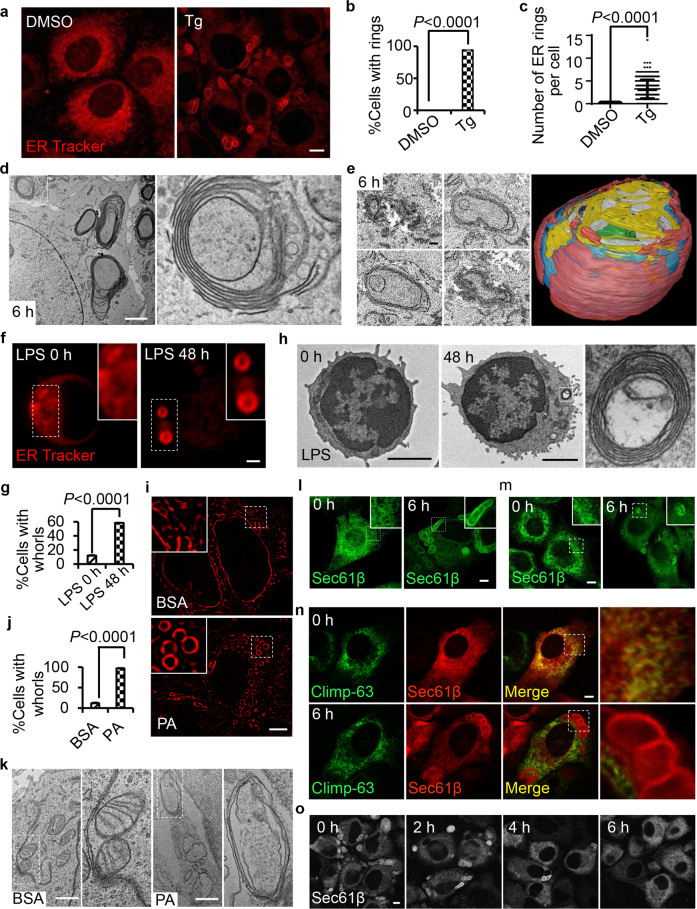
Formation of ER whorls in mammalian cells during ER stress. **a** NRK cells were treated with DMSO or 0.6 μM Tg for 6 h, and then stained with ER-Tracker Red and visualized by confocal microscopy. Scale bar, 10 μm. **b** Cells from **a** were quantified for % cells with ER rings (*n* = 3 independent experiments; >100 cells were assessed per independent experiment). Data represent means ± SE. **c** Cells from **a** were quantified for the number of ER rings per cell (*n* = 3 independent experiments; >100 cells were assessed per independent experiment). Data represent means ± SE. **d** TEM images of a NRK cell treated with Tg for 6 h. Scale bar, 2 μm. The right panel shows an enlarged image of the region of interest outlined in the left panel. **e** Cross-sectional FIB**-**SEM image analysis of ER whorls. Representative images (left) show individual cross-sections of the structure. Scale bar, 100 nm. Side view (right) of a 3D reconstruction from the aligned cross-sections showing the sphere-like structure. Different colors represent different layers. **f** B cells were purified from B6 splenocytes using MACS beads conjugated with anti-CD19 antibody and resuspended in culture medium. 2 × 10^6^ cells were cultured in the absence or presence of LPS (10 μg/mL) for 48 h, and then stained with ER-Tracker Red and visualized by Airyscan microscopy. Scale bar, 1 μm. Regions outlined with white dashed lines are magnified in the insets. **g** Cells from **f** were quantified for ER whorls (*n* = 3 independent experiments; >100 cells were assessed per independent experiment). Data represent means ± SE. **h** Representative TEM micrographs of cells from **f**. Scale bar, 2 μm. The boxed region is magnified on the right. **i** INS-1 cells were treated with BSA or PA for 6 h, and then stained with ER-Tracker Red and visualized by 3D-SIM microscopy (three-dimensional structured illumination microscopy). Scale bar, 5 μm. The region outlined with white dashed lines is magnified in the insets. **j** Cells from **i** were quantified for ER whorls (*n* = 3 independent experiments; >100 cells were assessed per independent experiment). Data represent means ± SE. **k** Representative TEM micrographs of cells from **i**. Scale bar, 1 μm. Regions outlined with wh**i**te dashed lines are magnified on the right. **l** GFP-Sec61β-expressing NRK cells were treated with Tg for 0 or 6 h and then observed by confocal microscopy. Scale bar, 5 μm. The region outlined with white dashed lines is magnified in the inset. **m** Immunostaining of endogenous Sec61β in NRK cells treated with Tg for 0 or 6 h. Scale bar, 5 μm. The region outlined with white dashed lines is magnified in the inset. **n** RFP-Sec61β-expressing NRK cells transfected with the sheet ER marker Climp-63-GFP were treated with Tg for 0 or 6 h and then observed by confocal microscopy. Regions of interest are boxed and magnified on the right. Scale bar, 5 μm. **o** NRK cells stably expressing GFP-Sec61β were treated with Tg for 6 h, and then Tg was withdrawn. Representative confocal images of the cells are shown at the indicated times after Tg withdrawal. Scale bar, 5 μm. *P* values were calculated using a two-tailed, unpaired *t*-test (**b**, **c**, **g**, **j**).

By serendipity, we found that thapsigargin (Tg) treatment can transform the ER into large ring-like structures. These ER rings have an elongated oval shape and their size can be up to 5 µm. In normal rat kidney (NRK) cells treated with Tg for 6 h, virtually every cell has at least one ER ring, and the average number of ER rings is 3 per cell (Fig. 1a–c). To better characterize these ER rings, we carried out transmission electron microscopy (TEM) analysis. Multiple concentric membrane structures were observed in Tg-treated cells. In these structures, multiple layers of membranes were stacked together, which is the characteristic feature of ER whorls (Fig. 1d). Under transmission electron microscope, ER whorls appear as 2-dimensional (2D) concentric ellipses, which can be interpreted as concentric circular ribbons or concentric spheres in 3 dimensions (3D). To determine the 3D structure of ER whorls, we carried out focused ion beam (FIB)‐SEM analysis. We found that ER whorls are sphere-like structures, with inner layers of continuous membrane that form a sphere and outer layers that dissolve into a heap of disheveled membrane structures on top (Fig. 1e).

Tg induced ER whorls in every cell line that we tested (Supplementary information, Fig. S1a). We also tested three other known ER stressors, dithiothreitol (DTT), tunicamycin (Tm) and cyclopiazonic acid (CPA), for their ability to induce ER whorls. Similar to Tg, CPA and DTT can induce ER whorl formation in a dose-dependent manner (Supplementary information, Fig. S1b–e). In contrast, even up to 50 µg/mL, Tm does not induce ER whorls (Supplementary information, Fig. S1d–g). We also tested ER whorl formation in two physiological UPR induction models, palmitic acid (PA)-induced ER stress in Ins-1 cells, and B cell differentiation.^15,16^ Importantly, ER whorls were observed in both of these physiologically relevant cellular models (Supplementary information, Fig. 1f–k), which indicates that ER whorl formation is a physiological response to ER stresses.

We found that ER whorls can be labeled by ectopically expressed full-length Sec61β, a subunit of the Sec61 ER translocon complex (Fig. 1l). Immunofluorescence analysis using a Sec61β antibody confirmed that endogenous Sec61β is recruited to ER whorls after Tg treatment (Fig. 1m). Overexpression of Sec61β protein does not enhance ER whorl formation (Supplementary information, Fig. S1h, i). These features make GFP-Sec61β a good marker for ER whorls. Next, we investigated whether other ER-resident proteins are also recruited to ER whorls. We co-transfected cells with Sec61β and markers for tubular ER (DP1, Rtn4a), sheet ER (Climp-63) and ER lumen (Calreticulin, KDEL), and we found that only Sec61β was recruited to ER whorls; moreover, these other ER-resident proteins still retained their typical ER pattern, which suggests that the ER co-exists with ER whorls (Fig. 1n; Supplementary information, Fig. S1j–m). Taken together, these data suggest that the ER is not transformed directly into ER whorls; rather, a specific set of ER constituents are compartmentalized into ER whorls, which co-exist with the rest of the ER.

ER whorls are induced by ER stressors, but do they disappear after ER stress is attenuated? We found that 6 h after withdrawal of Tg, all ER whorls disappeared (Fig. 1o). The disappearance of ER whorls is not due to macroautophagy or microautophagy, as ER whorls also disappear in ATG12 knockout (KO) and Chmp4b KO cells after withdrawal of Tg (Supplementary information, Fig. S1n–r). Thus, ER whorls are reversible structures which dynamically respond to strong ER stress.

### Formation of ER whorls is dependent on PERK activation

**Fig. 2:**
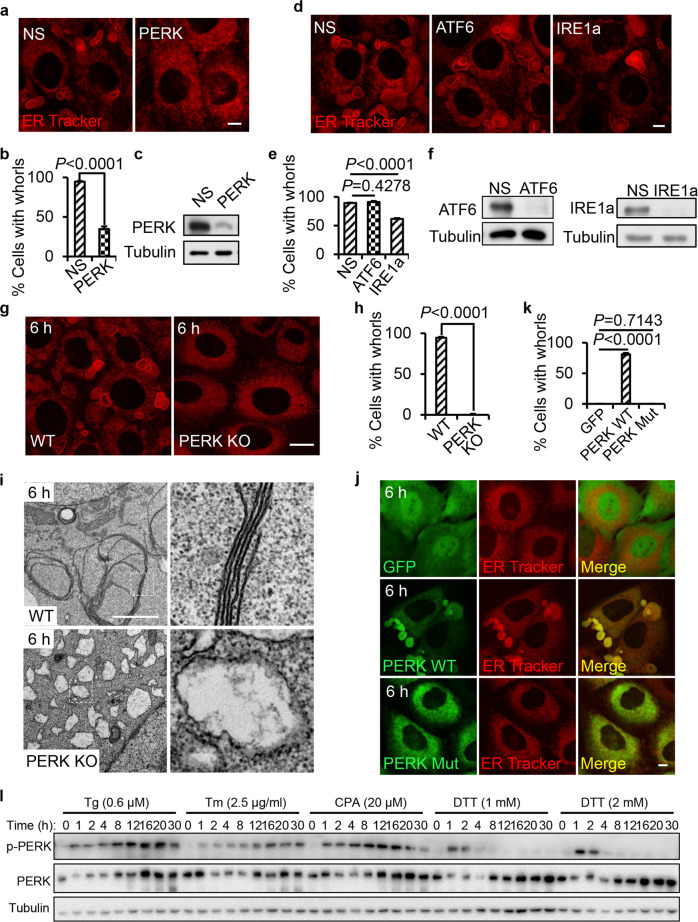
ER whorl formation is dependent on PERK activation. **a** NRK cells were transfected with nonspecific (NS) or PERK siRNA. Cells were treated with Tg for 6 h, and then stained with ER-Tracker Red and visualized by confocal microscopy. Scale bar, 5 μm. **b** Cells from **a** were quantified for ER whorls (*n* = 3 independent experiments; >100 cells were assessed per independent experiment). Data represent means ± SE. **c** PERK knockdown efficiency in cells from **a** was determined by western blot. **d** NRK cells were transfected with NS, ATF6, or IRE1a siRNA. Cells were treated with Tg for 6 h, and then stained with ER-Tracker Red and visualized by confocal microscopy. Scale bar, 5 μm. **e** Cells from **d** were quantified for ER whorls (*n* = 3 independent experiments; >100 cells were assessed per independent experiment). Data represent means ± SE. **f** Knockdown efficiency in cells from **d** was determined by western blot. **g** A PERK KO cell line was generated by CRISPR-Cas9. WT and PERK KO cells were treated with Tg for 6 h, and then stained with ER-Tracker Red and visualized by confocal microscopy. Scale bar, 10 μm. **h** Cells from **g** were quantified for ER whorls (*n* = 3 independent experiments; >100 cells were assessed per independent experiment). Data represent means ± SE. **i** Representative TEM micrographs of cells from **g**. Scale bar, 2 μm. Regions outlined with white dashed lines are magnified on the right. **j** PERK KO cells transfected with GFP, GFP-PERK, or GFP-PERK-Mut (K622A) were treated with Tg for 6 h and then observed by Opera Phenix microscopy with 60× confocal mode. Scale bar, 5 μm. **k** Cells from **j** were quantified for ER whorls (*n* = 3 independent experiments; >100 cells were assessed per independent experiment). Data represent means ± SE. **l** NRK cells were treated with Tg (0.6 μM), Tm (2.5 μg/mL), CPA (20 μM), DTT (1 mM) or DTT (2 mM) for the indicated times and analyzed by western blot. *P* values were calculated using a two-tailed, unpaired *t*-test (**b**, **e**, **h**, **k**).

Next, we investigated whether or not ER whorl formation is part of the UPR signaling pathway. We knocked down IRE1a, ATF6 and PERK, and we found that PERK knockdown markedly attenuated Tg-induced ER whorl formation (Fig. 2a–c). Knockdown of ATF6 did not affect ER whorl formation while IRE1a knockdown caused a small reduction of ER whorl formation (Fig. 2d–f). To further test the role of PERK in ER whorl formation, we generated PERK KO cells by CRISPR-Cas9. We found that Tg-, DTT- or CPA-induced ER whorl formation is completely blocked in PERK KO cells (Fig. 2g, h; Supplementary information, S2). TEM analysis confirmed that in PERK KO cells, the formation of ER whorls is completely blocked, and the ER swelled into large spherical structures (Fig. 2i). To test whether the kinase activity of PERK is required for ER whorl formation, we established two PERK KO cell lines in which wild-type (WT) or kinase-dead PERK was stably expressed. We found that WT PERK, but not the kinase-dead PERK, rescued the Tg-induced ER whorl formation (Fig. 2j, k). Thus, PERK kinase activity is required for ER whorl formation.

It is worth noting that there is a positive correlation between ER whorl formation and the strength and duration of PERK activation. Tm, which does not induce ER whorls, induces weak PERK activation. DTT, which induces a relatively low amount of ER whorls, only induces transient PERK activation. In contrast, Tg and CPA, which both induce higher levels of ER whorl formation, induce strong, prolonged PERK activation (Fig. 2l).

### ER whorls form from Sec61-containing tubular-vesicular precursors

**Fig. 3:**
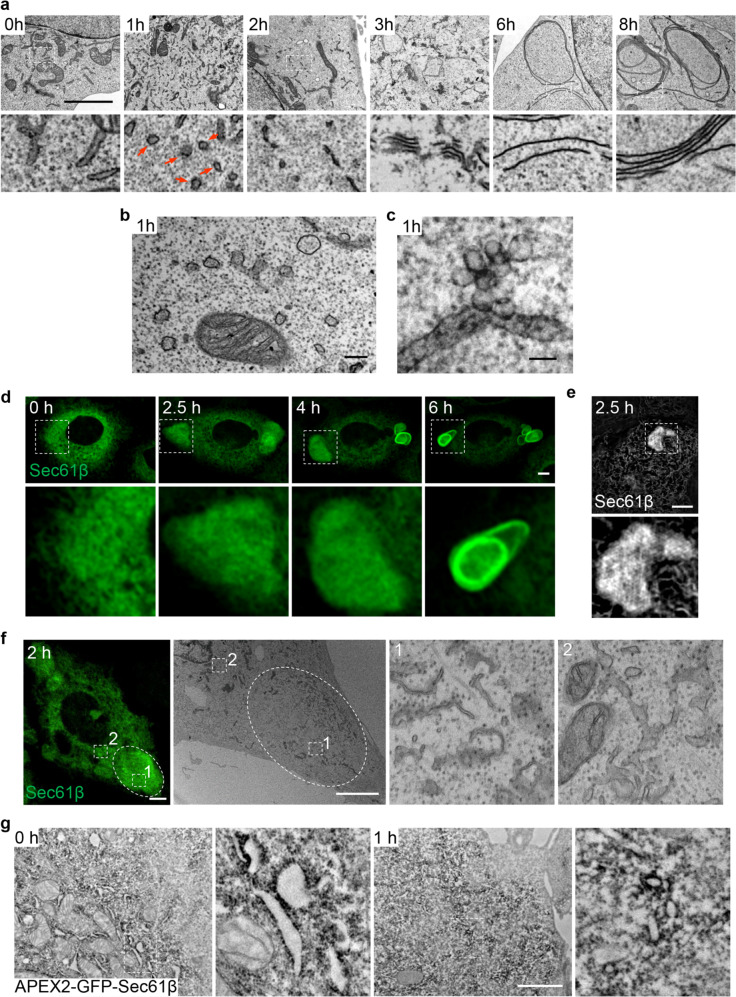
ER whorls are formed from Sec61-containing tubular-vesicular precursors. **a** Representative TEM micrographs of NRK cells treated with Tg for the indicated times. Regions of interest (outlined with white dashed lines) are magnified at the bottom. Red arrows in the lower 1 h panel indicate vesicles. Scale bar, 2 μm. **b** A TEM micrograph from **a** at 1 h. Scale bar, 200 nm. **c** A representative TEM micrograph of an NRK cell treated with Tg for 1 h. The block is processed to 300-nm-thick sections for observation. Scale bar, 100 nm. **d** NRK cells stably expressing GFP-Sec61β were treated with Tg and time-lapse images were acquired by Opera Phenix microscopy with 60× confocal mode. Regions of interest are magnified at the bottom. Scale bar, 5 μm. **e** NRK cells stably expressing GFP-Sec61β were treated with Tg for 2.5 h and visualized by GI-SIM at 100 nm resolution. Scale bar, 2 μm. The region of interest is magnified at the bottom. **f** CLEM imaging of a GFP-Sec61β-expressing NRK cell treated with Tg for 2 h. Left, the confocal image; middle, the TEM image; right, the enlarged images of the regions of interest outlined in the middle panel. 1, enlarged area of the GFP-Sec61β-condensed area; 2, enlarged area outside of the GFP-Sec61β-condensed area. Scale bar, 5 μm. **g** TEM image showing the DAB staining pattern in NRK cells stably expressing APEX2-GFP-Sec61β after treatment with Tg for 0 or 1 h. Scale bar, 2 μm.

To further characterize ER whorl formation, we carried out TEM analysis at different time points after Tg treatment. We found that the ER whorl biogenesis has several morphologically distinguishable stages. After 1 h of treatment, a large number of ribosome-free vesicles and tubules appeared (Fig. 3a). After 2 h of treatment, these tubular-vesicular structures started to align, and their lumens were visibly reduced. After 3 h, the lumens of these structures were completely lost, and multiple membranes started to stack together. After 6 h, ER whorls became visible, and after 8 h, ER whorl formation was complete (Fig. 3a). Detailed TEM analysis revealed that vesicles appeared to bud from the ER (Fig. 3b). We further carried out TEM analysis on 300-nm-thick blocks, in which more information in the z axis is preserved. Using this technique, we observed tubular-vesicular structures emerging from the ER, in which the vesicles are packed together like a bunch of grapes (Fig. 3c).

To test whether these tubular-vesicular structures are indeed the precursors of ER whorls, we carried out live-cell imaging of GFP-Sec61β. About 2 h after Tg treatment, the GFP-Sec61β signals started to condense, and ER whorls eventually emerged from this area of condensed GFP-Sec61β signal (Fig. 3d; Supplementary information, Movie S1). The region containing the condensed signal can be better appreciated by imaging using grazing incidence structured illumination microscopy (GI-SIM) (Fig. 3e). Next, we carried out correlative light-electron microscopy (CLEM) of GFP-Sec61β. CLEM revealed that the areas of condensed GFP-Sec61β signal are composed of a collection of vesicular-tubular structures (Fig. 3f), which are similar to those observed in standard TEM analysis. In 3D, these structures could be vesicles, tubules and small double-membrane sheets. As a control, we imaged areas outside the region containing the condensed GFP-Sec61β signal. The control areas appear to retain the normal ER morphology, with a larger radius for the tubular ER (Fig. 3f). Finally, we carried out APEX2-mediated proximity labeling on APEX2-GFP-Sec61β-expressing cells after 1 h of Tg treatment. APEX2 catalyzes the local deposition of DAB, which then binds electron-dense osmium, thus enhancing the contrast of EM images. We found that APEX2-GFP-Sec61β expressed well and was targeted to the correct location: it labeled ER whorls in Tg-treated cells (Supplementary information, Fig. S3a, b). We found that these vesicular-tubular structures in Tg-treated cells were indeed enriched with APEX2-GFP-Sec61β signals (Fig. 3g). Collectively, these data indicate that budding of Sec61β-positive tubular-vesicular ER whorl precursors occurs prior to formation of ER whorls.

### Recruitment of Sar1 initiates formation of ER whorl precursors

**Fig. 4:**
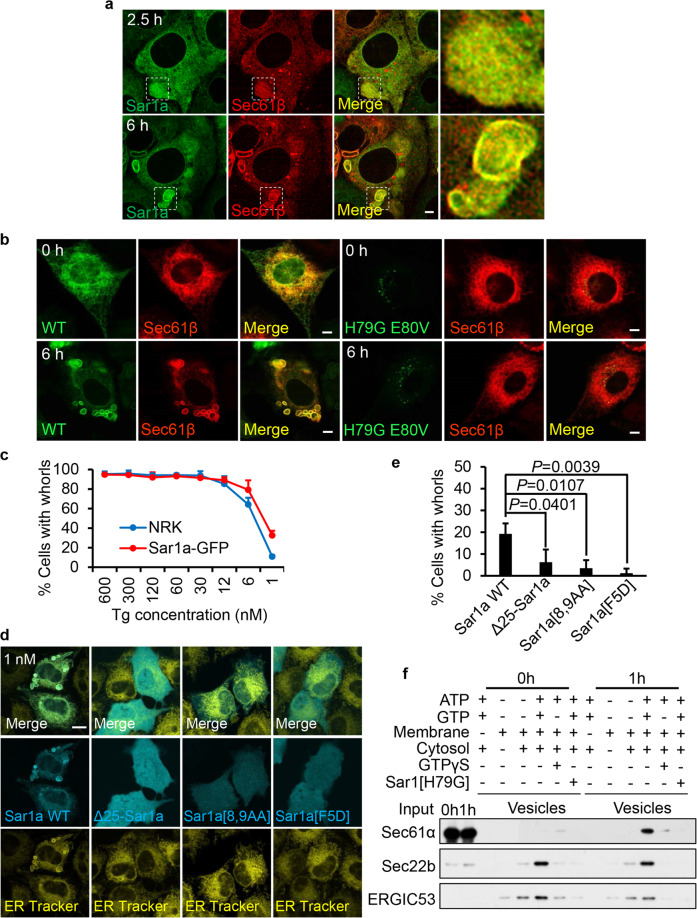
Recruitment of Sar1 initiates formation of ER whorl precursors. **a** Sar1a-GFP-expressing NRK cells transfected with RFP-Sec61β were treated with Tg for 2.5 or 6 h and then images were acquired by Opera Phenix microscopy with 60× confocal mode. Scale bar, 5 μm. The regions outlined with white dashed lines are magnified on the right (Merge). **b** RFP-Sec61β-expressing NRK cells were transfected with Sar1a-GFP (WT) or Sar1a[H79G]-GFP (H79G E80V). Cells were treated with Tg for 0 or 6 h, and then imaged with a confocal microscope. Scale bar, 5 µm. **c** NRK cells stably expressing Sar1a-GFP and NRK cells were treated with decreasing concentrations of Tg for 6 h, and then stained with ER-Tracker Red and visualized by confocal microscopy. Cells were quantified for ER whorls (*n* = 3 independent experiments; >100 cells were assessed per independent experiment). Data represent means ± SE. **d** NRK cells were transfected with Sar1a-GFP (Sar1a WT), Δ25-Sar1a-GFP, Sar1a[8,9AA]-GFP, or Sar1a[F5D]-GFP. Cells were treated with Tg (1 nM) for 6 h, and then stained with ER-Tracker Red and visualized by confocal microscopy. Scale bar, 10 μm. **e** Cells from **d** were quantified for ER whorls (*n* = 3 independent experiments; >50 cells were assessed per independent experiment). Data represent means ± SE. *P* values were calculated using a two-tailed, unpaired *t*-test. **f** Permeabilized NRK cells treated with Tg for 0 or 1 h were employed in an in vitro COPII budding assay. The resulting vesicle fractions and permeabilized cell input were separated by SDS-PAGE and visualized by immunoblotting.

The morphological similarity between ER whorl precursors and COPII vesicles prompted us to test the role of the COPII machinery in the formation of ER whorls. First, we carried out imaging of live cells expressing fluorescently-tagged COPII components including Sar1a-GFP, Sec13-GFP and Sec31A-GFP. We found that Sar1a-GFP is completely colocalized with Sec61β and concentrated on the ER whorl precursors and on ER whorls (Fig. 4a). In contrast, Sec13-GFP and Sec31A-GFP are not concentrated on ER whorl precursors or ER whorls (Supplementary information, Fig. S4a, b). In conventional COPII formation, Sar1 should disassociate from COPII vesicles shortly after vesicle formation. Therefore, the fact that Sar1 persistently localizes on ER whorl precursors indicates that ER whorl precursors are quite different from COPII vesicles.

It is known that overexpression of the constitutively active Sar1a[H79G] mutant can block cargo export from the ER by redistributing the ER export sites to the juxtanuclear membranes.^17^ We found that overexpression of this constitutively active mutant potently blocked ER whorl formation (Fig. 4b), Thus, Sar1 is involved in ER whorl formation.

The previous literature showed that Sar1 can directly deform liposomes into narrow membrane tubules, a process which depends on insertion of the N-terminal amphipathic α helix of Sar1 into the membrane.^18^ Replacing the bulky N-terminal hydrophobic residues with alanine diminished Sar1-mediated membrane tubulation. Interestingly, the morphology of the tubules induced by WT Sar1 is highly similar to that of the tubules we observed on ER whorl precursors and on ER whorls, raising the possibility that Sar1 may initiate the generation of ER whorl precursors by deforming the ER. We found that overexpressed Sar1 potentiated the induction of ER whorl formation by a very low dose (1 nM) of Tg (Fig. 4c). This observation allowed us to directly test whether the membrane-binding and tubulation capacity of Sar1 is required for formation of ER whorls. To do this, we generated the tubulation-incompetent mutants Δ25-Sar1a and Sar1a[8,9AA],^18^ and Sar1a[F5D], a mutant that cannot bind to membranes.^19^ We found that overexpression of each of these three mutants failed to potentiate the induction of ER whorl formation in the presence of 1 nM Tg (Fig. 4d, e). This suggests that Sar1-mediated membrane binding and tubulation are required for ER whorl formation.

We reasoned that budding of ER whorl precursors may share similar mechanisms with budding of COPII vesicles. Thus, we deployed the COPII budding assay to biochemically test the possible role of Sar1 in the formation of ER whorl precursors. For the membrane source, we used ER membranes from control cells or cells treated with Tg for 1 h.^20^ We found that in the untreated cells, Sec22b and ERGIC53 (both cargos for COPII vesicles) bud into vesicles in an ATP- and GTP-dependent manner (Fig. 4f). Adding GTPγS or a constitutively active Sar1 mutant (Sar1[H79G]) blocked the budding of Sec22b and ERGIC53, which indicated that the assay was working as expected. In the control membrane fraction, Sec61α, as demonstrated in the literature, did not bud into COPII vesicles.^3^ In contrast, in the Tg-treated membrane fraction, Sec22b, ERGIC53 and Sec61α budded into vesicles in an ATG- and GTP-dependent manner, and similarly the budding of Sec22b and Sec61α was blocked by GTPγS and Sar1[H79G] (Fig. 4f). Collectively, these data suggest that ER stress can induce Sar1-dependent budding of vesicles from the ER, which include ER-resident proteins that are not usually sorted into COPII vesicles.

### COPII machinery and Sec22 are required for ER whorl formation

**Fig. 5:**
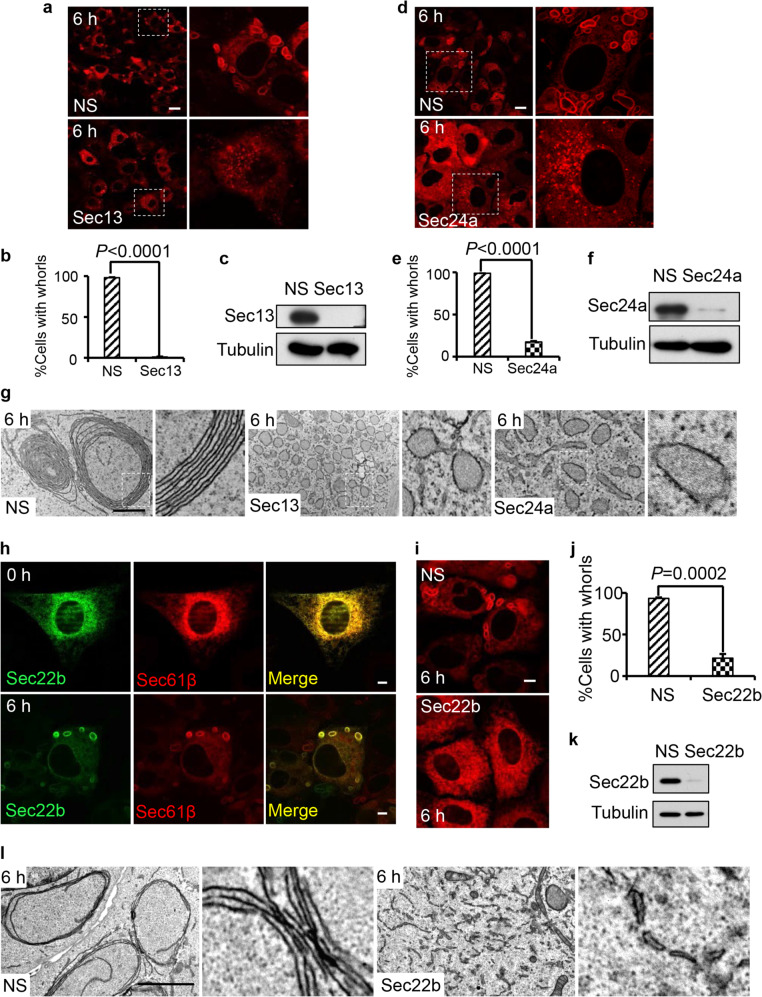
COPII machinery and Sec22b are required for ER whorl formation. **a** RFP-Sec61β-expressing NRK cells were transfected with NS or Sec13 siRNA. Cells were treated with Tg for 6 h. Scale bar, 20 μm. Regions outlined with white dashed lines are magnified on the right. **b** Cells from **a** were quantified for ER whorls (*n* = 3 independent experiments; >100 cells were assessed per independent experiment). Data represent means ± SE. **c** Sec13 knockdown efficiency in cells from **a** was determined by western blot. **d** RFP-Sec61β-expressing NRK cells were transfected with NS or Sec24a siRNA. Cells were treated with Tg for 6 h. Scale bar, 10 μm. Regions outlined with white dashed lines are magnified on the right. **e** Cells from **d** were quantified for ER whorls (*n* = 3 independent experiments; >100 cells were assessed per independent experiment). Data represent means ± SE. **f** Sec24a knockdown efficiency in cells from **d** was determined by western blot. **g** Representative TEM micrographs of cells from **a** and **d**. Scale bar, 1 μm. Regions outlined with white dashed lines are magnified. **h** RFP-Sec61β-expressing NRK cells transfected with GFP-Sec22b were treated with Tg for 0 or 6 h and then observed by confocal microscopy. Scale bar, 5 μm. **i** RFP-Sec61β-expressing NRK cells were transfected with NS or Sec22b siRNA. Cells were treated with Tg for 6 h. Scale bar, 5 μm. **j** Cells from **i** were quantified for ER whorls (*n* = 3 independent experiments; >100 cells were assessed per independent experiment). Data represent means ± SE. **k** Sec22b knockdown efficiency in cells from **i** was determined by western blot. **l** Representative TEM micrographs of cells from **i**. Scale bar, 2 μm. Regions outlined with white dashed lines are magnified. *P* values were calculated using a two-tailed, unpaired *t*-test (**b**, **e**, **j**).

The Sar1 dependency of ER whorl formation suggested that other components of the COPII machinery may also be involved in ER whorl formation. To test the role of the COPII machinery in formation of ER whorls, we knocked down Sec13, a component of the outer coat of COPII. We also knocked down Sec24a, a component of the COPII inner coat. We found by confocal microscopy and TEM analyses that knocking down Sec13 or Sec24a blocked the formation of ER whorl precursors and ER whorls (Fig. 5a–f); instead, the ER swelled into spherical structures with ribosomes still attached (Fig. 5g). Taken together, these data suggest that COPII machinery is required for budding of ER whorl precursors.

To form an ER whorl, ER whorl precursors must go through multiple rounds of fusion. To determine whether the SNAREs are required for ER whorl formation, we screened known ER-localized SNAREs. We reasoned that if a SNARE is required for ER whorl formation, it may localize on ER whorls. Among five ER-localized SNAREs tested, we found that Sec22b, Bet1 and Gosr2/GS27 were localized on ER whorls (Fig. 5h; Supplementary information, Fig. S5a). Next, we knocked down all three of these ER whorl-localized SNAREs individually, and found that knockdown of Sec22b blocked ER whorl formation (Fig. 5i–k). TEM analysis showed that knockdown of Sec22b caused a different phenotype compared to knockdown of components of the COPII machinery. In cells with knockdown of Sec13 or Sec24a, the ER swelled into large spherical vesicles with ribosomes still attached (Fig. 5g). In contrast, knockdown of Sec22b did not affect the budding of ER whorl precursors; however, the vesicles did not fuse and, as a result, a large amount of ribosome-free ER whorl precursors accumulated inside Sec22b-knockdown cells (Fig. 5l; Supplementary information, Fig. S5b). These data indicate that Sec22b is likely required for fusion of ER whorl precursors into ER whorls.

### COPII machinery is required for PERK activation

**Fig. 6:**
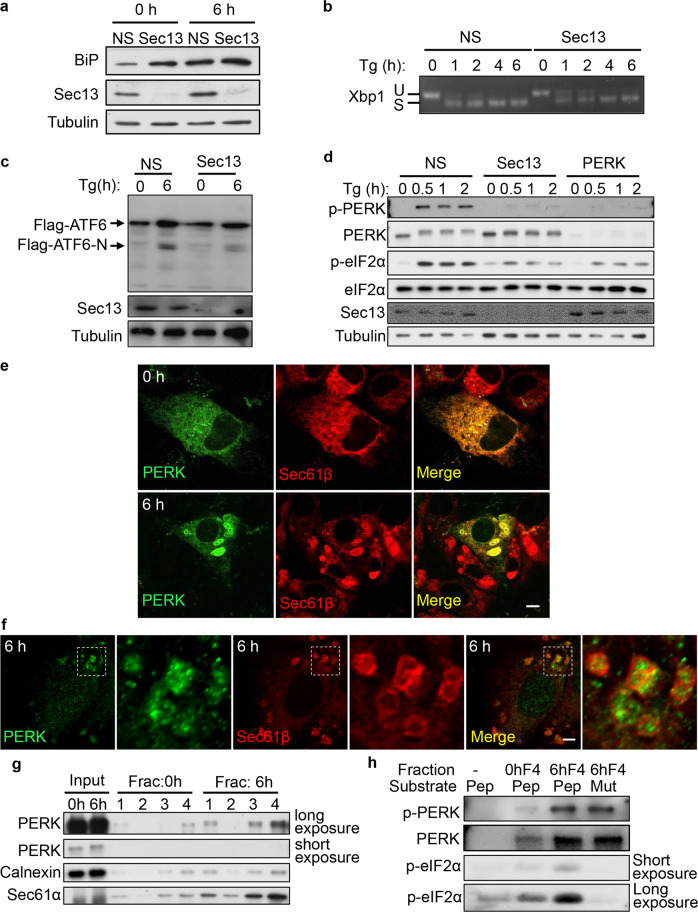
COPII machinery is required for PERK activation. **a** NRK cells were transfected with NS or Sec13 siRNA. Cells were treated with Tg for 0 or 6 h and analyzed by western blot using an antibody against BiP. **b** NRK cells were transfected with NS or Sec13 siRNA, and treated with Tg for the indicated times. Total RNA was subsequently extracted for semiquantitative RT-PCR analysis of XBP1 mRNA species (Xbp1 S: spliced XBP1 mRNA band; Xbp1 U: unspliced XBP1 mRNA band). **c** Flag-ATF6-expressing NRK cells were transfected with NS or Sec13 siRNA. Cells were treated with Tg for 0 or 6 h and analyzed by western blot using an antibody against Flag. **d** NRK cells were transfected with NS or Sec13 siRNA. Cells were treated with Tg for the indicated times and analyzed by western blot using an antibody against phospho-PERK (Thr980) and phospho-eIF2α (Ser51). **e** RFP-Sec61β-expressing NRK cells transfected with GFP-PERK were treated with Tg for 0 or 6 h and then observed by confocal microscopy. Scale bar, 5 μm. **f** Immunostaining of endogenous PERK and RFP in RFP-Sec61β-expressing NRK cells treated with Tg for 6 h. Scale bar, 5 μm. Regions outlined with white dashed lines are magnified. **g** NRK cells treated with Tg for 0 or 6 h were homogenized and the lysates were subjected to 1000× *g* centrifugation to discard the nuclei. The supernatant was ultracentrifuged in OptiPrep density gradient medium. The distribution of PERK in the fractions was monitored by western blot. 1 is the top fraction. **h** In vitro protein kinase assays. Membranes were collected from Fraction 4 of NRK cells treated with Tg for 6 h as described in **g** and mixed with ATP and the peptides eIF2α p(45–56) or mutant eIF2α p(45–56, S51A). Kinase reactions were resolved on SDS-PAGE and visualized by immunoblotting using an antibody against phospho-eIF2α (Ser51).

Next, we investigated the roles of COPII machinery in the UPR. First, we assessed the role of COPII machinery in upregulation of BiP, which operationally defines the UPR. We found that knockdown of Sec13 markedly enhances the BiP expression in both control and Tg-treated cells (Fig. 6a), which indicates a higher ER stress load in these cells. Next, we tested the role of COPII in each branch of the UPR. We found that knockdown of Sec13 caused a mild reduction of XBP1 processing (Fig. 6b), and it reduced ATF6 activation (Fig. 6c), which is known to require COPII-mediated ER-to-Golgi trafficking. In contrast, Sec13 knockdown strongly blocked the activation of PERK, and also reduced the PERK-mediated phosphorylation of eIF2α at Ser51 (Fig. 6d). Similarly, knockdown of Sec24a reduced the activation of PERK and the PERK-mediated phosphorylation of eIF2α (Supplementary information, Fig. S6a). In contrast, knockout of Sec22b did not block the activation of PERK or the PERK-mediated phosphorylation of eIF2α (Supplementary information, Fig. S6b). These data suggest that formation of ER whorl precursors, but not the formation of ER whorls, facilitates ER stress-induced PERK activation.

We wondered whether or not PERK itself can be sorted into ER whorls. We found that GFP-PERK is indeed translocated to ER whorls after Tg treatment (Fig. 6e). Similarly, we found that endogenous PERK is sorted into ER whorls (Fig. 6f). We then carried out membrane fractionation assays on control and Tg-treated cells. We found that after Tg treatment, PERK and Sec61α were enriched in fraction 4 of Tg-treated cells (Fig. 6g). However, calnexin, which does not localize on ER whorls (Supplementary information, Fig. S6c), was not highly enriched in fraction 4. Together, these findings indicate that ER whorls are enriched in fraction 4. Furthermore, fraction 4 was capable of phosphorylating an established PERK substrate peptide, eIF2α P(45–56), at Ser51 (Fig. 6h). These results confirmed that ER whorls contain activated PERK. Collectively, we conclude that PERK is sorted into ER whorls after the onset of ER stress, and the COPII machinery is required for PERK activation induced by Tg treatment.

### Segregation of translocons into translation-incompetent ER whorls

**Fig. 7:**
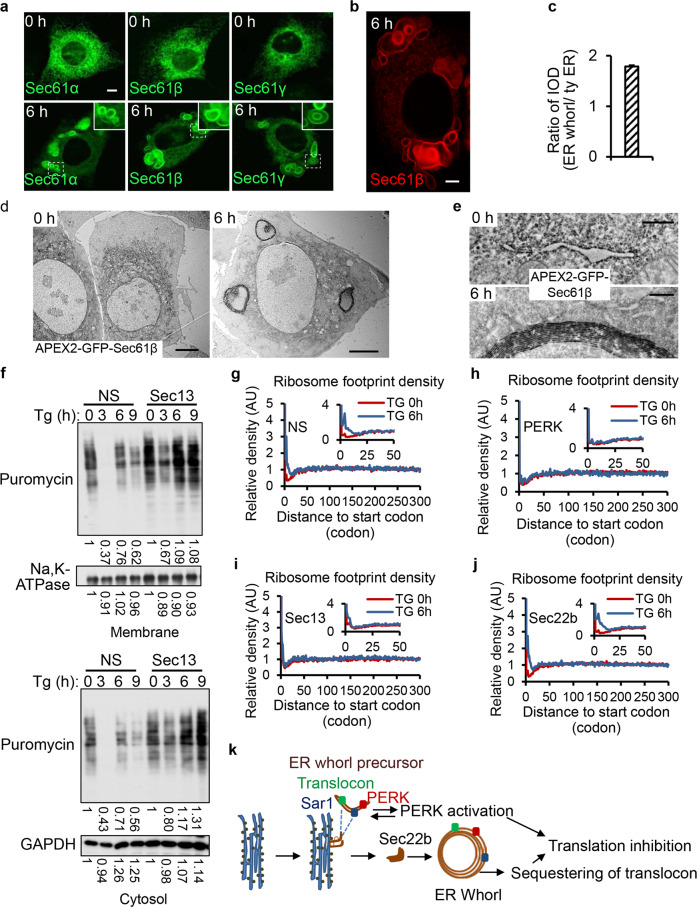
COPII machinery is required for ER stress-induced inhibition of protein translation. **a** All three translocon components (GFP-Sec61α, GFP-Sec61β, GFP-Sec61γ) localize on ER whorls in cells treated with Tg for 6 h. Regions of interest are outlined with white dashed lines and magnified in the insets. Scale bar, 5 μm. **b** RFP-Sec61β-expressing NRK cells were treated with Tg for 6 h and visualized by 2D-confocal microscopy. Scale bar, 5 μm. **c** Cells from **b** were analyzed for IOD (integrated optical density) of ER whorls and the rest of the ER by Image-Pro plus (*n* = 3 independent experiments; >100 cells were assessed per independent experiment). Data represent means ± SE. **d** TEM images showing the DAB staining pattern in NRK cells stably expressing APEX2-GFP-Sec61β after treatment with Tg for 0 or 6 h. Scale bar, 5 μm. **e** Representative images of APEX2-GFP-Sec61β-labeled structures in cells from **d**. Scale bar, 500 nm. **f** NRK cells were transfected with NS or Sec13 siRNAi. Cells were treated with Tg for the indicated times and incubated with puromycin for 30 min. Membrane proteins and cytosolic proteins were separately detected by immunoblotting using an anti-puromycin antibody. **g**–**j** Average density of ribosome footprints on all coding genes aligned by their start codons. The analyses were done in NRK cells treated with NS (**g**), PERK siRNA (**h**), Sec13 siRNA (**i**), or Sec22b siRNA (**j**), before (0 h) and after (6 h) Tg treatment. The ribosome footprints were allocated to each codon according to their P-sites, and the count of footprints on each codon was normalized by the average count per codon after the first 30 codons. **k** A model of ER whorl formation and function.

Sec61β is a component of the translocon complex, which is essential for importing nascent peptides into the ER lumen. One striking feature of ER whorls is that they do not have ribosomes attached (Fig. 1d), and consequently are translationally incompetent. Sorting of translocons into ribosome-free ER whorls thus may represent an additional mechanism of translational inhibition. To test this hypothesis, we examined whether other translocon components are also sorted into ER whorls, and we found that indeed all three translocon subunits are enriched in ER whorls (Fig. 7a). To analyze the relative distribution of Sec61β in ER whorls and on regular ER, we carried out 2D imaging of GFP-Sec61β. We found that the majority of Sec61β signal is present on ER whorls (Fig. 7b, c). The enrichment of Sec61β in ER whorls was further verified by APEX2-based intracellular-specific protein imaging by electron microscopy.^21^ More detailed analysis confirmed that ER with normal morphology still exists in the Tg-treated cells; however, APEX2-GFP-Sec61β signals are no longer localized on the ER but are concentrated on ER whorls (Fig. 7d, e). These data confirmed that the majority of Sec61β is sorted into ER whorls from the ER. Taken together, these data imply that sustained ER stress triggers segregation of translocons into translation-incompetent ER whorls, thus likely contributing to the attenuation of ER protein import.

### COPII machinery is required for ER stress-induced inhibition of protein translation

The facts that COPII machinery is required for PERK activation, and translocons are sequestered in the translation-incompetent ER whorls, prompted us to test whether COPII machinery is required for ER stress-induced inhibition of protein translation. Puromycin is a Tyr-tRNA mimetic that enters the ribosome A site and terminates translation by ribosome-catalyzed covalent incorporation into the C-terminus of the nascent chain. Thus, translation can be measured by detection of puromycylated nascent chains released from ribosomes by immunoblotting using anti-puromycin antibody.^22^ We labeled Tg-treated cells with puromycin, and then isolated the cytosol and membrane fractions to assess new protein synthesis using an anti-puromycin antibody. We found that knockdown of Sec13 indeed partially alleviated the ER stress-induced translation inhibition of both membrane and cytosolic proteins (Fig. 7f).

To validate this observation, we used ribosome profiling to quantitatively assess the protein translation changes that occur upon ER stress. The quality of our ribosome profiling experiments was underscored by the observation that ribosome-protected fragments (RPFs) were highly enriched in the annotated CDS regions and showed strong 3-nt periodicity based on their P-site positions. Next, we assessed genome-wide RPFs mapped on the CDS regions, aligned by their start codons. We found that Tg treatment resulted in a significant increase of ribosome occupancy in the first ~30 codons (Fig. 7g). Such a phenomenon has been frequently reported before as a signature of translation inhibition in response to stresses,^23^ amino acid starvation,^24^ and inhibition of known translation factors.^25^ We found that knockdown of PERK abolished the increase of ribosome occupancy in the first ~30 codons (Fig. 7h), which suggested that cells lacking PERK also lack ER stress-induced translation inhibition. Similarly, in Sec13-knockdown cells, Tg treatment no longer induced significant accumulation of ribosome occupancy at the 5′ end of the mRNAs (Fig. 7i). In contrast, knockdown of Sec22b did not affect the global enrichment of the ribosome footprints in the first 30 codons after Tg treatment (Fig. 7j). Note that knockdown of Sec13 or Sec22b in the absence of Tg treatment did not result in any noticeable global shift of the ribosome footprint densities (Supplementary information, Fig. S7). These results showed that depletion of Sec13 largely attenuated the responses of the cells to Tg treatment in terms of translation elongation, whereas Sec22b-depleted cells retained the translation elongation defect in response to ER stress induced by Tg treatment. Taken together, our data indicate that formation of ER whorl precursors seems to be required for ER stress-induced translation inhibition.

## Discussion

In this study, we investigated the biogenesis, formation mechanism and possible function of ER whorls. We found that ER whorls can be induced by a set of ER stressors, and formation of ER whorls is dependent on PERK activity and mediated by the COPII machinery. Exposure to these ER stressors can induce budding of GFP-Sec61β-positive vesicular-tubular structures from the ER. The budding of these structures is so extensive that they form a densely packed mesh which is visible under the confocal microscope. Despite the fact that formation of the vesicular-tubular structures requires COPII machinery, these vesicular-tubular structures show different characteristics from COPII vesicles. For example, Sar1 is persistently localized on them. These vesicular-tubular structures eventually transform into ER whorls in a process that depends on the ER SNARE Sec22b. Functionally, COPII is required for ER stress-induced inhibition of protein translation, possibly by modulating PERK activity and sequestering translocons in ribosome-free ER whorls (Fig. 7k).

The relationship between COPII, PERK, ER whorl precursors and ER whorls is quite complicated. There are 4 components in this pathway, COPII, PERK, ER whorl precursors and ER whorls. Our data show that COPII makes ER whorl precursors which then transform into ER whorls; thus, the causal link between COPII, ER whorl precursors and ER whorls is relatively clear. On the other hand, there is no causal link between PERK activation and formation of ER whorls, as Sec22b knockdown blocks ER whorl formation but does not block PERK activation. The relationship between formation of ER whorl precursors and PERK activation is a classic example of reciprocal causation, which cannot be defined by a simple hierarchical relationship. Our study found that COPII contributes to PERK activation. At this point, we do not know how COPII is linked to PERK activation. However, it is well established that oligomerization and trans-autophosphorylation of PERK are required for PERK activation. Therefore, one interesting hypothesis is that sorting PERK into ER whorl precursors and ER whorls by COPII may concentrate PERK into a small area, thus promoting the oligomerization and trans-autophosphorylation of PERK. It is worth noting that this hypothesis does not suggest that formation of ER whorl precursors is required for initiation of PERK activation; it is more likely that COPII-mediated formation of ER whorl precursors amplifies the activation process. Further investigation is needed to address this question.

At present, the precise sequence of events of ER whorl formation is still not completely clear. The time-lapse imaging and CLEM show that ER whorl formation is preceded by the appearance of a densely packed meshwork formed by Sec61β- and Sar1a-positive vesicular-tubular structures. The observations that these structures are positive for both Sec61β and Sar1a, and ER whorls always emerge from inside the meshwork, suggest that these vesicular-tubular structures are ER whorl precursors. At this point, we are not able to directly image the fusion of ER whorl precursors into ER whorls. The ER whorl precursors are small and densely packed together, and cannot be observed even with GI-SIM. Nevertheless, since the ER SNARE Sec22b is localized on ER whorls and Sec22b knockdown causes accumulation of vesicular-tubular structures and blocks ER whorl formation, it is highly plausible that ER whorls may form through Sec22b-mediated fusion of ER whorl precursors.

In yeast, ER whorls can be induced by ER stressors, and they are engulfed by vacuoles through ESCRT-dependent selective autophagy. It is worth noting that there are some key differences between ER whorls in yeast and in mammals. First, in yeast, ER whorls can be induced by Tm, while Tm fails to induce ER whorls in mammalian cells. Second, the sizes are very different: in yeast, the size of ER whorls is around 200 nm,^17^ while in mammalian cells, it can be up to 10 µm. Whether or not yeast and mammalian ER whorls share similar biogenesis mechanisms remains to be investigated.

How does the UPR signaling pathway regulate ER whorl formation? Formation of ER whorls depends on PERK activation. In PERK KO cells, the generation of ER whorl precursors is blocked and the ER swells into large spherical structures. ER whorl formation is also mediated by COPII machinery, and knockdown of components of the COPII machinery also blocks the formation of ER whorl precursors and causes swelling of the ER. Thus, both PERK and the COPII machinery are required for budding of ER whorl precursors from the ER. It is possible that PERK directly phosphorylates components of the COPII machinery, which triggers the modulation of secretory pathways and generation of ER whorl precursors; alternatively, PERK may phosphorylate a subset of cargo proteins which are not normally sorted into COPII vesicles; these proteins may then nucleate the cargo sorting process for generation of ER whorl precursors. Future investigations are needed to dissect the detailed mechanism of how PERK regulates ER whorl formation.

Our study shows that Sar1 is persistently localized on ER whorl precursors and is eventually concentrated in ER whorls. This contrasts with the conventional COPII vesicles, from which Sar1 rapidly disassociates after formation. The previous literature showed that Sar1 can cause liposome tubulation, and the morphology of these Sar1-induced membrane tubules is similar to that of the tubular ER whorl precursors we observed. Thus, it is likely that ER whorl formation is initiated by recruitment of Sar1 to the ER, which transforms a patch of the ER into a network of narrow tubules and vesicles. Moreover, unlike the budding process of conventional COPII vesicles, Sar1 is permanently associated with ER whorl precursors, and thus keeps the precursors in a tubular form for fusion into ER whorls.

We found that the COPII machinery is required for PERK activation, which controls the general inhibition of protein translation. Furthermore, the sorting of the protein translocon into ER whorl precursors and ER whorls, which lack ribosomes, will shut down the translation of membrane and secreted proteins. These mechanisms are likely to ease the unfolded protein load in the ER and reduce secretion. We propose that, under ER stress conditions, the COPII machinery actively turns from a pathway for secretion to a pathway for controlling protein translation, thus reducing secretion and protein translation simultaneously.

ER whorl formation is reversible once the ER stressor is removed. We speculate that ER whorls may also function as a reservoir for ER proteins whose functions are important for the house-keeping roles of the ER but are not required for the ER stress response, similar to the formation of stress granules during various stress responses. In this scenario, proteins are sorted into ER whorls during the ER stress response; however, when the ER stress is attenuated, these proteins can return to the ER, thus rapidly restoring the proper function of the ER.

Another important question is what determines the specific sorting of proteins that are destined to become localized in ER whorls. The Sec61 complex is not sorted into COPII vesicles in cells grown in normal conditions; however, shortly after Tg treatment, Sec61 complex is sorted into the unconventional COPII vesicles in a COPII-dependent manner. Thus, a mechanism must exist for recognizing and sorting ER stress-specific cargo proteins. It is possible that Sec24a, the cargo adapter for COPII, acquires the ability to bind these cargo molecules through post-translational modification, or new adapters may be involved that still remain to be identified.

## Materials and methods

### Cell culture

NRK cells were cultured in DMEM medium (Hyclone; SH30022.01) supplemented with 10% fetal bovine serum (FBS), 2 mM Glutamax and penicillin-streptomycin. Tg (Sigma) treatment was at 0.1–0.6 μM, DTT (Sigma) treatment was at 1 or 2 mM, CPA (Sigma) was used at 20 μM, Tm (Sigma) was used at 2.5 μg/mL for 6 h unless otherwise stated.

### B cell differentiation

B cells were purified from B6 splenocytes using MACS beads conjugated with anti-CD19 antibody and resuspended in culture medium. 2 × 10^6^ cells were cultured in the absence or presence of LPS (10 μg/mL, Sigma) for 48 h, and then stained with the dye ER-Tracker Red and visualized by Airyscan microscopy (Zeiss) at 120 nm resolution.

### INS-1 cell treatment

INS-1 cells were cultured in RPMI 1640 medium containing 10% FBS, 1 mM sodium pyruvate, 50 μM β-mercaptoethanol (β-ME), 100 U/mL penicillin, and 100 μg/mL streptomycin in a humidified atmosphere with 5% CO_2_ at 37 °C. Palmitate was dissolved at 100 mM in 0.1 M sodium hydroxide to make a stock solution. The palmitate stock solution was diluted in culture medium to which fatty acid-free BSA had been added, in a 1:19 molar ratio of palmitate to fatty acid-free BSA, to prepare BSA-conjugated palmitate. Cells were incubated with the BSA-conjugated palmitate at 0.5 mM for 6 h, and then stained with ER-Tracker Red and visualized by 3D-SIM at 120 nm resolution.

### Transfection

NRK cells were transfected with 2 µg DNA or 200 pmol siRNA via Amaxa nucleofection^TM^ using program X-001 and nucleofector solution according to the manufacturer’s instructions.

### Generation of NRK cells with stable knockout of Sec22b and PERK

The CRISPR/Cas9 system was a gift from Dr. Wei Guo (Tsinghua University). The sgRNA-binding sequence (5′-GTCCCGCCTCAGTGCGGCGGA-3′) was used to disrupt Sec22b. The sgRNA-binding sequence (5′-GTTCTGATTATACTGGCTGG-3′) was used to disrupt PERK. gRNA, Cas9 and puromycin plasmids were co-transfected into NRK cells. After 24 h, cells were cultured in medium containing puromycin (1 µg/mL) for ~7 days. Monoclones were picked and transferred into 48-well plates for amplification and analyses by western blot and DNA sequencing.

### APEX2 stable cell lines

Lentivirus particles were collected from the supernatant of HEK293T cells 2 days after co-transfection of APEXS-GFP-Sec61β constructs with the lentivirus packaging plasmids VSV-G, and dR8.91 (the APEX2 system was a gift from Dr. Peng Zou). Transfection was performed with PEI (Polyscience). The supernatant was purified using a 0.45 μm filter. NRK cells were transduced with purified lentivirus particles and selected with 8 μg/mL blasticidin for 7 days.

### Antibodies

Antibodies were used at the indicated dilutions as follows: anti-Sec61α (Cell Signaling Technology, Cat# 14868, 1:1000), anti-Sec61β (Abcam, ab15576, 1:1000), anti-Sec22b (Synaptic Systems, Cat# 186003, 1:2500), anti-ERGIC53 (Sigma, E1031, 1:2000), anti-Sec13 (Santa Cruz Biotech, sc-103196, 1:1000), anti-Sec24a (gift from Xiaowei Chen, 1:500), anti-S6 (Cell Signaling Technology, Cat# 2317, 1:1000), anti-puromycin (Millipore, MABE342, 1:1000), anti-Na, K-ATPase (Cell Signaling Technology, Cat# 3010, 1:1000), anti-GAPDH (Proteintech, 60004-1-Ig, 1:1500), anti-PERK (Cell Signaling Technology, Cat# 3192, 1:1000), anti-Phos-PERK (Cell Signaling Technology, Cat# 3179, 1:1000), anti-Calnexin (Abcam, ab22595, 1:1000), anti-eIF2α (Cell Signaling Technology, Cat# 5324, 1:1500), anti-phos-eIF2α (Cell Signaling Technology, Cat# 3398, 1:1000), anti-tubulin (Zen-BioScience, Cat# 200608, 1:8000), Goat Anti-Mouse IgG1, Human ads-HRP (SouthernBiotech, Cat# 1070-05, 1:5000), Goat Anti-Rabbit IgG, Human ads-HRP (SouthernBiotech, Cat# 4010-05, 1:5000) and Rabbit Anti-Goat IgG (H + L)-HRP (SouthernBiotech, Cat# 6160-05, 1:3500).

### Western blot

One plate (35 mm dish) of NRK cells grown to ~80%–90% confluency were lysed with 0.6 mL of lysis buffer (2% SDS, 50 mM Tris-HCl (pH 6.8), 2 mM EDTA (pH 8.0), 1 mM PMSF (Sigma) and complete EDTA-free protease inhibitor cocktail (Roche)). The lysates were mixed with 4× loading buffer and heated at 95 °C for 10 min. Samples were separated by 10%/12%/15% SDS-PAGE and transferred onto nitrocellulose membrane (GE Company) or PVDF membrane (Millipore). The membranes were blocked in TBS-T (20 mM Tris-HCl, pH 7.6, 137 mM NaCl and 0.1% Tween-20) containing 3% BSA at room temperature for 1 h. Primary antibodies were diluted in Solution I Buffer (TOYOBO). The membranes were incubated with primary antibodies at 4 °C overnight. After incubation, the membranes were washed three times with TBS-T and then incubated with horseradish peroxidase (HRP)-conjugated secondary antibodies diluted in TBS-T containing 3% BSA at room temperature for 1 h. After final washes with TBS-T, the membranes were developed using Westar ETA C 2.0 (CYANAGEN) and exposure to X-ray film (Kodak).

### Immunofluorescence

Cells were grown on Lab-Tek Chambered cover glasses (NUNC), and fixed with 4% paraformaldehyde (PFA) in PBS buffer at room temperature for 10 min, permeabilized with 0.1% saponin for 10 min, and then blocked with 10% goat serum for 1 h. Incubation with antibody was performed in 10% goat serum in PBS for 1 h at room temperature. The primary antibody used was rabbit anti-Sec61β (Abcam, ab78276, 1:300), or mouse IgG1 anti-PERK (Santa Cruz, sc-377400, 1:100). The secondary antibody used was Goat anti-Rabbit IgG (H + L) Cross-Adsorbed Secondary Antibody, Alexa Fluor 488 (Thermo Fisher, A-11008, 1:500) or Goat anti-Mouse IgG (H + L) Cross-Adsorbed Secondary Antibody, Alexa Fluor 488 (Thermo Fisher, A32723, 1:500).

### Confocal microscopy

Immunofluorescence microscopy was performed using an Olympus FV-1000 confocal microscope (Olympus, Japan) equipped with a UPlanSApo 60×/1.35 oil immersion objective.

For live-cell imaging, images were acquired with an Olympus FV-1000 confocal microscope equipped with a Chamlide environmental incubator system (CU-109), which maintained the NRK cells at 37 °C and 5% CO_2_.

### Opera Phenix microscopy with 60× confocal mode

High content live-cell imaging: 30,000 NRK cells were seeded into a 96-well olefin-bottom imaging plate (Perkin Elmer, Cat# 6055302). The plate was placed in a pre-heated (37 °C) Opera Phenix microscope with a 60× water-immersion lens (Perkin Elmer) at 5% CO_2_. Images were acquired at 1080 × 1080 pixels using Harmony software.

### TEM

Cells were cultured on 35-mm dishes and fixed in 2.5% glutaraldehyde (GA; SPI, 02607-BA) in 0.1 M sodium phosphate buffer (pH 7.2) for 2 h at room temperature. The cells were washed in the same buffer three times, post-fixed in 1% osmium tetroxide (Ted Pella)/1.5% K_3_[Fe(CN)_6_] in distilled water for 1 h at 4 °C, and then dehydrated with a graded ethanol series (50%, 70%, 80%, 90%, 100%, 100%) for 2 min each on ice. The cells were rinsed once at room temperature to avoid condensation, infiltrated in Pon 812 (SPI) using 1:1 (v/v) anhydrous ethanol and resin for 30 min, 1:2 for 30 min, 1:3 for 30 min, 100% resin for 1 h, fresh resin for 1 h, and then polymerized in an oven at 60 °C for 48 h. Ultrathin sections of 70 nm were cut by ultramicrotome (Leica EM UC7). After staining with uranyl acetate and lead citrate, sections were observed under a transmission electron microscope with 80 kV (Hitachi, H-7650).

### CLEM

Gridded glass-bottom dishes (Cellvis, D35-14-1.5GI) were used to culture GFP-Sec61β-expressing NRK cells. Cells were fixed with 2% PFA and 2.5% GA first, and then a spinning-disk confocal microscope was immediately used to collect 4–6 bright field and confocal images to document the cells at different magnifications. After that, the cells were dehydrated with a graded ethanol series (50%, 70%, 90%, 95%, and 100%) for 8 min each. Samples were infiltrated and embedded in SPON12 resin. After polymerization for 48 h at 60 °C, 70-nm-thick ultrathin sections were cut using a diamond knife, and then picked up with Formvar-coated copper grids (100 mesh). The sections were double stained with uranyl acetate and lead citrate. After air drying, samples were examined with a transmission electron microscope (H-7650) at an acceleration voltage of 80 kV.

### APEX2 for TEM

Cells with stable expression of APEX2-GFP-Sec61β or Sec13-APEX2-GFP were cultured in 35-mm dishes. The same volume of 37 °C pre-warmed 2.5% GA in SC buffer (100 mM sodium cacodylate with 2 mM CaCl_2_, pH 7.4) was added to the dish. After 5 min incubation at room temperature, the medium was replaced with fresh 2.5% GA for 5 min, and the dishes were quickly moved to ice. The following steps were all on ice. After 45 min incubation, cells were rinsed with cold SC buffer for 3 × 5 min, incubated in SC buffer containing 20 mM glycine (to quench unreacted GA) for 5 min, and then rinsed again in SC buffer for 3 × 5 min. Cells were incubated in a freshly prepared solution of 0.5 mg/mL (1.4 mM) DAB tetrahydrochloride (Sigma, D5637) in SC buffer for 2 min and then changed to a fresh solution containing 0.5 mg/mL (1.4 mM) DAB and 0.003% (v/v) (1 mM) H_2_O_2_ for 1–5 min. Then the DAB solution was removed, and the cells were rinsed for 3 × 5 min with chilled SC buffer. Post-fixation staining was performed with 2% osmium tetroxide for 5 min. Cells were rinsed for 3 × 5 min in distilled water, and then placed in 2% aqueous uranyl acetate (Electron Microscopy Sciences) overnight. The samples were then dehydrated in a graded ethanol series (50%, 70%, 80%, 90%, 100%, 100%) for 2 min each on ice. After a final incubation in 100% ethanol for 2 min at room temperature, the cells were infiltrated and embedded in Pon 812 resin. DAB-stained areas of embedded cells were identified by transmitted light, and the areas of interest were cut out using a razor blade and mounted on resin blocks with cyanoacrylic adhesive. The blocks were trimmed, and 70-nm ultrathin sections were cut, and then examined by transmission electron microscope (Hitachi, H7650).

### FIB‐SEM

For FIB-SEM, cells were exposed on the resin surface and a layer of gold (~20 nm thick) was deposited on the cells. On the FIB-SEM (FEI, Helios Nano Lab 600i), a layer of platinum (~600 nm thick) was deposited on a surface perpendicular to the block face to be imaged. The block face was imaged using an electron beam with 2 keV acceleration voltage, 0.4 nA beam current, and 10 μs/pixel dwell time. After the block face was imaged, a gallium ion beam with an acceleration voltage of 30 keV and a current of 0.79 nA was used to mill the 10-nm-thick superficial layer from the block face for the next round of imaging and milling. After the entire volume was acquired, the images were imported into Amira software and aligned. The cellular compartments were manually traced and annotated using Amira v7.0 software (https://www.amira.com/).

### In vitro budding assay

Two plates (150 mm dishes) of NRK cells grown to ~80%–90% confluency were treated with Tg or not for 1 h. Cells were collected by trypsinization and sedimented at 200× *g* for 5 min at 4 °C. After resuspension in B88 buffer (20 mM HEPES, pH 7.2, 250 mM sorbitol, and 150 mM KOAc) and permeabilization with 40 µg/mL digitonin on ice for 4 min, ice-cold B88 buffer was added to cells to stop the permeabilization. Permeabilized cells were sedimented at 1000× *g* for 5 min at 4 °C. After two washes in B88 buffer to remove the cytosol, permeabilized cells were resuspended in 0.4 mL B88 buffer. Budding reactions (200 µL) were assembled on ice with 40 µL semi-intact cells as donor membranes, mouse liver cytosol at 4 mg/mL final concentration, 20 µL of 10× ATP regeneration system (10 mM ATP, 2 mg/mL creatine phosphokinase, 400 mM creatine phosphate, 2 mg/mL creatine phosphokinase, and 5 mM MgOAc in B88 buffer) and 4 µL of 10 mM GTP. Reactions were performed at 30 °C for 45 min and stopped by centrifuging at 14,000× *g* for 15 min at 4 °C. 180 µL supernatant was centrifuged in a TLA100 rotor at 131,440× *g* for 20 min at 4 °C. The supernatants were discarded, and the pellets were washed once and thoroughly resuspended in 20 µL of 2% SDS lysis buffer. After heating at 55 °C for 20 min, the lysates were mixed with 4× SDS loading buffer supplemented with 10% β-ME and heated at 95 °C for 10 min before SDS-PAGE.

### XBP1 mRNA splicing assay

Total RNA was isolated from cells using an RNeasy Mini Kit (Qiagen) and 1 µg of total RNA was reverse transcribed to cDNA with a Reverse Transcription System (Promega). 2 μL of cDNA was mixed with 0.2 mM of forward and reverse primers (Rat_XBP1_Fwd: 5′-TGGCCGGGTCTGCTGAGTCCG-3′; Rat_XBP1_Rev: 5′-ATCCATGGGAAGATGTTCTGG-3′), 0.2 mM of each dNTP, and 0.5 units of Taq DNA polymerase (Thermo Scientific). The reaction was set at an annealing temperature of 60.5 °C with an extension time of 30 s for 26 cycles. The products were then visualized on a 3% agarose gel (comprised of a 1:1 mixture of low-melting point agarose and standard agarose) stained by 1:10,000 SybrSAFE.

### ER purification

ER purification was performed using an ER isolation kit (Sigma-Aldrich, ER0100) according to the manufacturer’s instructions with slight modifications. More than 4 plates (150 mm dishes) of NRK cells grown to ~80%–90% confluency were treated with Tg or not for 6 h. Cells were collected by scraping, pooled and centrifuged at 1000× *g* for 5 min at 4 °C. After two washes in ice-cold PBS, cells were resuspended in a volume of 1× Hypotonic Extraction buffer (100 mM HEPES, pH 7.8, 10 mM EGTA and 250 mM potassium chloride) equivalent to 3 times the packed cell volume and incubated on ice for 30 min. Swollen cells were sedimented at 600× *g* for 5 min at 4 °C and resuspended in a volume of 1× Isotonic Extraction buffer (50 mM HEPES, pH 7.8, 1.25 M sucrose, 5 mM EGTA, and 125 mM potassium chloride) with protease inhibitors and phosphatase inhibitors equivalent to 2 times the packed swollen cell volume. The cells were broken with over 200 strokes of a Dounce homogenizer and the lysed cells were centrifuged at 1000× *g* for 10 min at 4 °C. The supernatant was diluted to 20% Optiprep solution (60% solution of iodixanol in water) and subjected to centrifugation at 150,000× *g* for 4 h in an Optiprep gradient composed of 30%, 20%, 15% and 10%. After centrifugation, fractions (360 µL/fraction) were collected and analyzed by western blot and TEM. All procedures were conducted at 4 °C.

### In vitro kinase assay

The fourth fraction was collected as described above from NRK cells treated with Tg for 0 or 6 h. Fraction 4 was diluted in PBS, and centrifuged at 20,000× *g*. The pellet was diluted with 50 µL 1× kinase buffer (50 mM Tris-HCl, pH 7.5, 10 mM MgCl_2_, 1 mM EGTA, 1 mM DTT and 0.01% Brij35). Reactions were assembled on ice with 10 µL resuspended pellet, 3 µg of peptide of WT eIF2α p(45–56) or mutant eIF2α p(45–56, S51A), and 5 mM ATP. Reactions were performed at 30 °C for 45 min and stopped by adding 4× SDS sample buffer.

### Puromycin labeling of newly synthesized cytosolic and membrane proteins

NRK cells (6-well plate) grown to 40%–50% confluency were treated with 1 µM puromycin for 30 min under normal culture conditions. After that, cytosolic and membrane proteins were extracted using a ProteoExtract native membrane protein extraction kit (M-PEK, Millipore) according to the manufacturer’s instructions.

### Purification of RPFs and total RNA for ribosome profiling

About 5 × 10^6^ cells were washed with prechilled PBS (containing 100 μg/mL cycloheximide, Sigma-Aldrich) and then harvested by centrifugation at 4 °C (2000 rpm, 5 min). The cell pellet of each sample was resuspended in prechilled lysis buffer (20 mM Tris-HCl, pH 7.4, 150 mM NaCl, 5 mM MgCl_2_, 0.1% NP-40, 1% Triton X-100, 1 mM DTT, 25 U/mL of RNase-free Dnase I, and 100 µg/mL cycloheximide). After 10 min of incubation on ice with periodic agitation, the lysate was centrifuged for 10 min (20,000× *g* at 4 °C), and the supernatant was collected. 400 µL of the clarified lysate was used for purification of the RPFs for ribosome profiling, and 200 µL of the lysate was used for total RNA sequencing in parallel.

The libraries for parallel ribosome profiling and total RNA sequencing were prepared with a protocol adapted from previously published methods.^26,27^ For RPFs, 90 Units of RNase I (Life Technologies, AM2294) for each A260 of the lysate was added to 200 µL of the supernatant and incubated at room temperature for 45 min with gentle mixing. Nuclease digestion was stopped with 15 µL of SUPERase In^TM^ RNase Inhibitor (Life Technologies, AM2696) and chilled on ice. Ribosome-RNA complexes were purified by Sephacryl S400 spin column chromatography (GE Healthcare, 27514001). The ribosome-protected RNA was extracted with Trizol (Invitrogen, 15596018), following the manufacturer’s protocol. Next, the ribosomal RNA was depleted using the RiboZero kit (Illumina; MRZH11124), following the manufacturer’s protocol. The samples were then resolved in a 15% urea gel by electrophoresis, and the area containing fragments of 25–35 nucleotides was excised from the gel. The RNA fragments were finally eluted for at least 2 h in 400 µL nuclease-free water, 40 µL of 5 M ammonium acetate (Invitrogen, AM9070G) and 2 µL of 10% SDS (Invitrogen, AM9823), and then precipitated in isopropanol (Sigma Aldrich, I9030).

Total RNA from the tissue samples was isolated from 100 µL of the clarified tissue lysate with Trizol (Invitrogen, 15596018), and the ribosomal RNA was then depleted using the RiboZero kit (Illumina; MRZH11124). Next, the rRNA-depleted total RNA was fragmented with PNK buffer (NEB, M0201L) at 95 °C for 20 min.

### Sequencing library preparation

Both RPFs and fragmented total RNA were cloned and amplified for next-generation sequencing with a tagging-based workflow. In brief, the RNA fragments were end-repaired with T4 PNK (NEB, M0201L) and ligated with a 3′ adapter, followed by cDNA synthesis, cDNA gel purification, circularization and PCR amplification.^26^ The sequencing libraries were assessed with a BioAnalyzer and quantified using a KAPA SYBR FAST Universal qPCR Kit (Kapa Biosystems, KK4601) prior to and after pooling for sequencing. Library insert sizes were typically about 30 bp. The pooled libraries were sequenced on the Illumina HiSeq 2500 platform with a single-end sequencing strategy for 50 cycles.

### Processing of the ribosome profiling and RNA-seq data

The pre-processing procedure of the ribosome profiling data and the parallel RNA-seq data has been described previously.^28,29^ Specifically, the 3′ adapters were trimmed from the raw reads of both mRNA and RPF.^30^ Low-quality reads with Phred quality scores lower than 20 (> 50% of bases) were removed using the fastx quality filter (http://hannonlab.cshl.edu/fastx_toolkit/). The reads originating from rRNAs were identified and discarded by aligning the reads to rat rRNA sequences (5S, 5.8S, 18S, and 28S) using Bowtie (version 1.1.2) with no mismatch allowed. The remaining reads were then mapped to the rat genome and spliced transcripts using STAR with the following parameters: –outFilterType BySJout –outFilterMismatchNmax 2 –outSAMtype BAM –quantMode TranscriptomeSAM –outFilterMultimapNmax 1 –outFilterMatchNmin 16. To control the noise from multiple alignments, reads mapped to multiple genomic positions were discarded.

### Analysis of gene differential translation efficiencies

The bioinformatics pipeline Xtail was used for quantitative and systematic analyses of the differential translation efficiencies.^28^ Preparation of the data for Xtail has been described previously. Specifically, the mRNA expression was estimated from the RNA-seq reads, which were counted using HTSeq-count (version 0.7.2).^28,31^ The RPF reads were subjected to multiple steps of read filtering, which reduced the technical noise of ribosome profiling and extracted the reads originating from ribosome-binding and translating sequences in coding regions. First, RPF reads with lengths of 25–35 nt were deemed high quality and most likely to be from ribosome occupation in mammalian cells.^32,33^ Second, reads with multiple alignments were discarded, and only the reads uniquely mapped to the coding regions were counted for RPFs. Third, due to the potential accumulation of ribosomes around the starts and ends of coding regions, reads aligned to the first 30 and last 5 codons were excluded for the counting of RPFs. Finally, the RNA and RPF read counts were processed by the Xtail package for genome-wide assessment of the differential translation efficiencies.^28^

### Reagent and resource sharing

Further information and requests for resources and reagents should be directed to and will be fulfilled by the lead contact, Li Yu.

## Supplementary information

Supplementary information, Figure S1

Supplementary information, Figure S2

Supplementary information, Figure S3

Supplementary information, Figure S4

Supplementary information, Figure S5

Supplementary information, Figure S6

Supplementary information, Figure S7

Supplementary information, Movie S1

Supplementary movie legend
