# Supplementary material for: COPII mitigates ER stress by promoting formation of ER whorls
Source: Cell Res. 2020 Sep 28;31(2):141–56. doi: 10.1038/s41422-020-00416-2 (PMC8026990; doi:10.1038/s41422-020-00416-2)
Supplement: Supplementary file 1 — Supplementary information, Figure S1 [file 41422_2020_416_MOESM1_ESM.pdf]

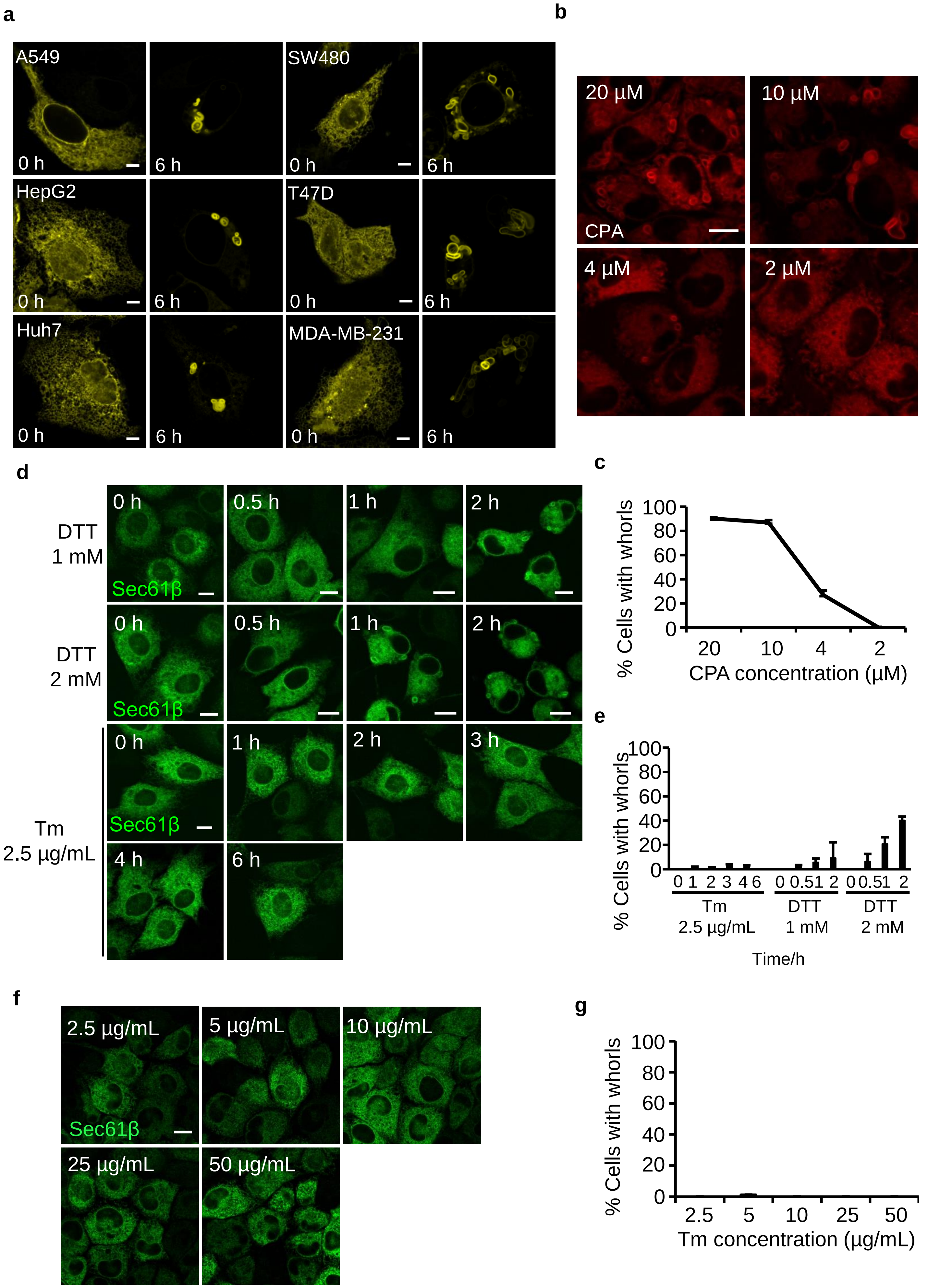
a
A549
0 h
6 h
SW480
0 h
6 h
HepG2
0 h
6 h
T47D
0 h
6 h
Huh7
0 h
6 h
MDA-MB-231
0 h
6 h
b
20 µM
10 µM
CPA
4 µM
2 µM
c
% Cells with whorls
100
80
60
40
20
0
20
10
4
2
CPA concentration (µM)
d
DTT
1 mM
0 h
0.5 h
1 h
2 h
Sec61β
DTT
2 mM
0 h
0.5 h
1 h
2 h
Sec61β
Tm
2.5 µg/mL
0 h
1 h
2 h
3 h
Sec61β
4 h
6 h
e
% Cells with whorls
100
80
60
40
20
0
0 1 2 3 4 6
0 0.51 2
0 0.51 2
Tm
2.5 µg/mL
DTT
1 mM
DTT
2 mM
Time/h
f
2.5 µg/mL
5 µg/mL
10 µg/mL
Sec61β
25 µg/mL
50 µg/mL
g
% Cells with whorls
100
80
60
40
20
0
2.5
5
10
25
50
Tm concentration (µg/mL)

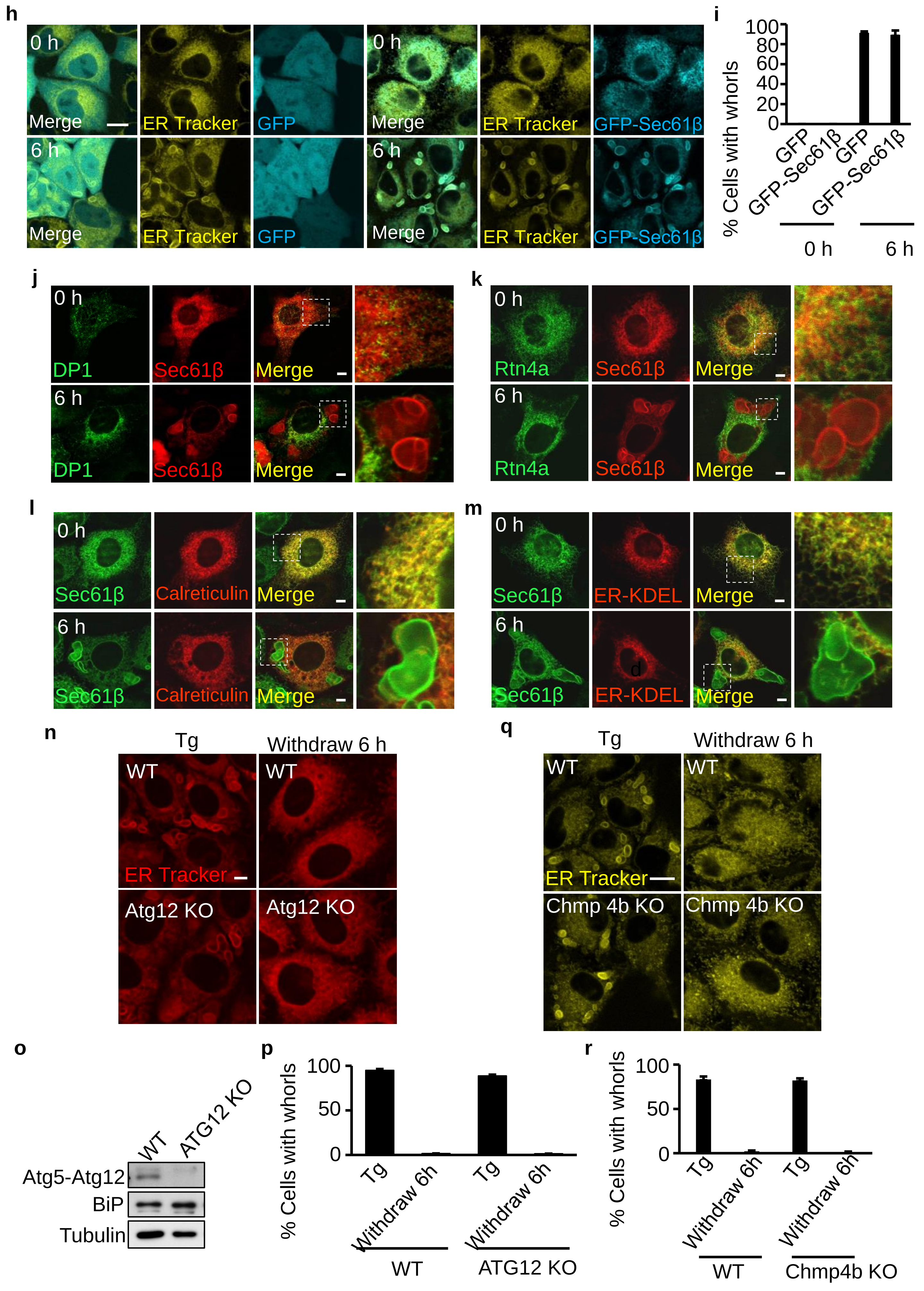

h
0 h
Merge
ER Tracker
GFP
GFP-Sec61β
6 h
i
% Cells with whorls
100
80
60
40
20
0
GFP
GFP-Sec61β
0 h
6 h
j
DP1
Sec61β
Merge
k
Rtn4a
l
Calreticulin
m
ER-KDEL
n
Tg
Withdraw 6 h
WT
Atg12 KO
q
Chmp 4b KO
o
ATG12 KO
Atg5-Atg12
BiP
Tubulin
p
50
Withdraw 6h
r
Chmp4b KO

Supplementary information, Fig. S1 a A549 (human lung carcinoma), SW480 (human adenocarcinoma), HepG2 (human hepatocellular carcinoma), T47D (human breast cancer), Huh7 (human well differentiated hepatocellular carcinoma cells), and MDA-MB-231 (human breast cancer) cells were transfected with RFP-Sec61β, treated with Tg or not for 12 h, and then observed by confocal microscopy. Scale bar, 5 μm. **b** NRK cells were treated with cyclopiazonic acid (CPA) at the indicated concentrations for 6 h, stained with ER-Tracker Red, and then visualized by confocal microscopy. Scale bar, 10 μm. **c** Cells from **b** were quantified for ER whorls (n = 3 independent experiments; more than 100 cells were assessed per independent experiment). Data represent means ± SE. **d** GFP-Sec61β-expressing NRK cells were treated with dithiothreitol (DTT) or tunicamycin (Tm) at the indicated concentrations and times, and then observed by confocal microscopy. Scale bar, 10 μm. **e** Cells from **d** were quantified for ER whorls (n = 3 independent experiments; more than 100 cells were assessed per independent experiment). Data represent mean ± SE. **f** GFP-Sec61β-expressing NRK cells were treated with tunicamycin (Tm) for 6 h at the indicated concentrations, and then observed by confocal microscopy. Scale bar, 10 μm. **g** Cells from **f** were quantified for ER whorls (n = 3 independent experiments; more than 50 cells were assessed per independent experiment). Data represent means ± SE. **h** NRK cells transfected with GFP or GFP-Sec61β were treated with Tg for 0 or 6 h, stained with ER-Tracker Red, and then visualized by confocal microscopy. Scale bar, 10 μm. **i** Cells from **h** were quantified for ER whorls (n = 3 independent experiments; more than 100 cells were assessed per independent experiment). Data represent means ± SE. **j, k** RFP-Sec61β-

expressing NRK cells transfected with the tubular ER marker GFP-DP1 or Rtn4a-GFP were treated with Tg for 0 or 6 h and then observed by confocal microscopy. Regions of interest are outlined with white dashed lines and magnified to the right. Scale bar, 5 μm. **l, m** GFP-Sec61β-expressing NRK cells transfected with the lumenal ER marker RFP-Calreticulin or ER-DsRed were treated with Tg for 0 or 6 h and then observed by confocal microscopy. Regions of interest are outlined with white dashed lines and magnified to the right. Scale bar, 5 μm. **n** NRK wild-type (WT) cells and Atg12-knockout (Atg12 KO) cells were treated with Tg for 6 h, and then Tg was withdrawn. Representative confocal images of the cells stained with the dye ER-Tracker Red are shown after Tg treatment and 6 h after Tg withdrawal (withdraw 6 h). Scale bar, 10 μm. **o** Knockout efficiency of cells from **n** was determined by western blot. **p** Cells from **n** were quantified for ER whorls (n = 3 independent experiments; more than 100 cells were assessed per independent experiment). Data represent means ± SE. **q** NRK WT cells and Chmp4b-knockout (Chmp4b KO) cells were treated with Tg for 6 h, and then Tg was withdrawn. Representative confocal images of the cells stained with the dye ER-Tracker Red are shown after Tg treatment and 6 h after Tg withdrawal (withdraw 6 h). Scale bar, 10 μm. **r** Cells from **q** were quantified for ER whorls (n = 3 independent experiments; more than 100 cells were assessed per independent experiment). Data represent means ± SE.
