# Supplementary material for: COPII mitigates ER stress by promoting formation of ER whorls
Source: Cell Res. 2020 Sep 28;31(2):141–56. doi: 10.1038/s41422-020-00416-2 (PMC8026990; doi:10.1038/s41422-020-00416-2)
Supplement: Supplementary file 2 — Supplementary information, Figure S2 [file 41422_2020_416_MOESM2_ESM.pdf]

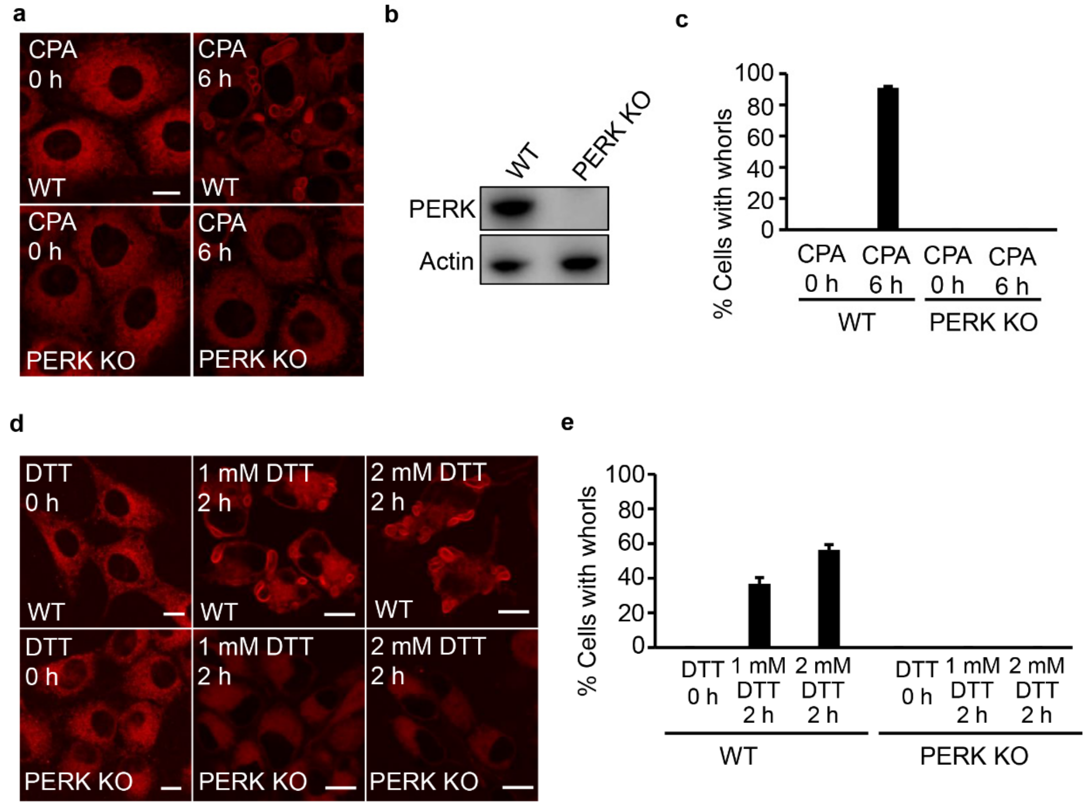

Supplementary information, Fig. S2 **a** A PERK-knockout (KO) cell line was generated by CRISPR-Cas9. Wild-type (WT) and PERK KO cells were treated with CPA (20 μM) for 6 h and then stained with ER-Tracker Red and visualized by Opera Phenix microscopy with 60× confocal mode. Scale bar, 10 μm. **b** Knockout efficiency of cells from **a** was determined by western blot. **c** Cells from **a** were quantified for ER whorls (*n* = 3 independent experiments; more than 100 cells were assessed per independent experiment). Data represent means ± SE. **d** WT and PERK KO cells were treated with DTT at the indicated concentration and time, stained with ER-Tracker Red, and then visualized by confocal microscopy. Scale bar, 10 μm. **e** Cells from **d** were quantified for ER whorls (*n* = 3 independent experiments; more than 100 cells were assessed per independent experiment). Data represent means ± SE.
