# Supplementary material for: COPII mitigates ER stress by promoting formation of ER whorls
Source: Cell Res. 2020 Sep 28;31(2):141–56. doi: 10.1038/s41422-020-00416-2 (PMC8026990; doi:10.1038/s41422-020-00416-2)
Supplement: Supplementary file 3 — Supplementary information, Figure S3 [file 41422_2020_416_MOESM3_ESM.pdf]

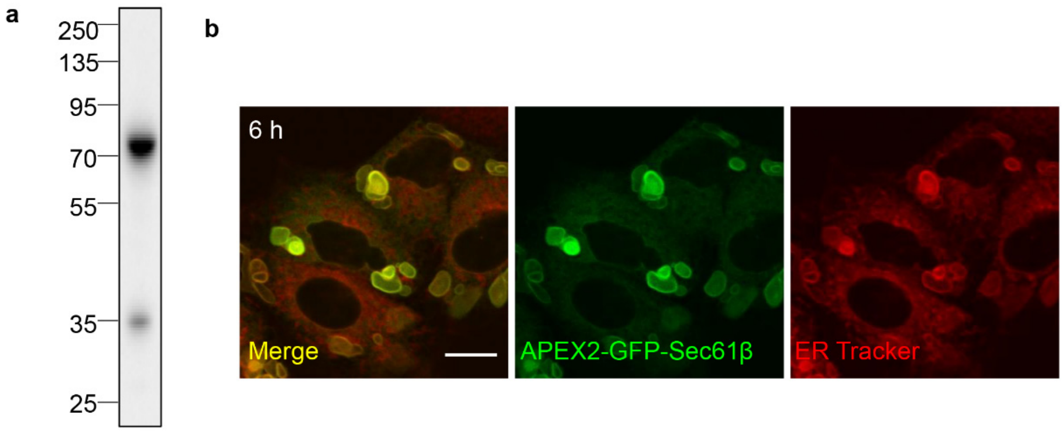


**Supplementary information, Fig. S3 a** NRK cells stably expressing APEX2-GFP-Sec61β were harvested and analyzed by western blot using anti-GFP antibody. **b** Cells from **a** were treated with 0.6 μM Tg for 6 h, stained with ER-Tracker Red, and then visualized by confocal microscopy. Scale bar, 10 μm.
