# Supplementary material for: COPII mitigates ER stress by promoting formation of ER whorls
Source: Cell Res. 2020 Sep 28;31(2):141–56. doi: 10.1038/s41422-020-00416-2 (PMC8026990; doi:10.1038/s41422-020-00416-2)
Supplement: Supplementary file 4 — Supplementary information, Figure S4 [file 41422_2020_416_MOESM4_ESM.pdf]

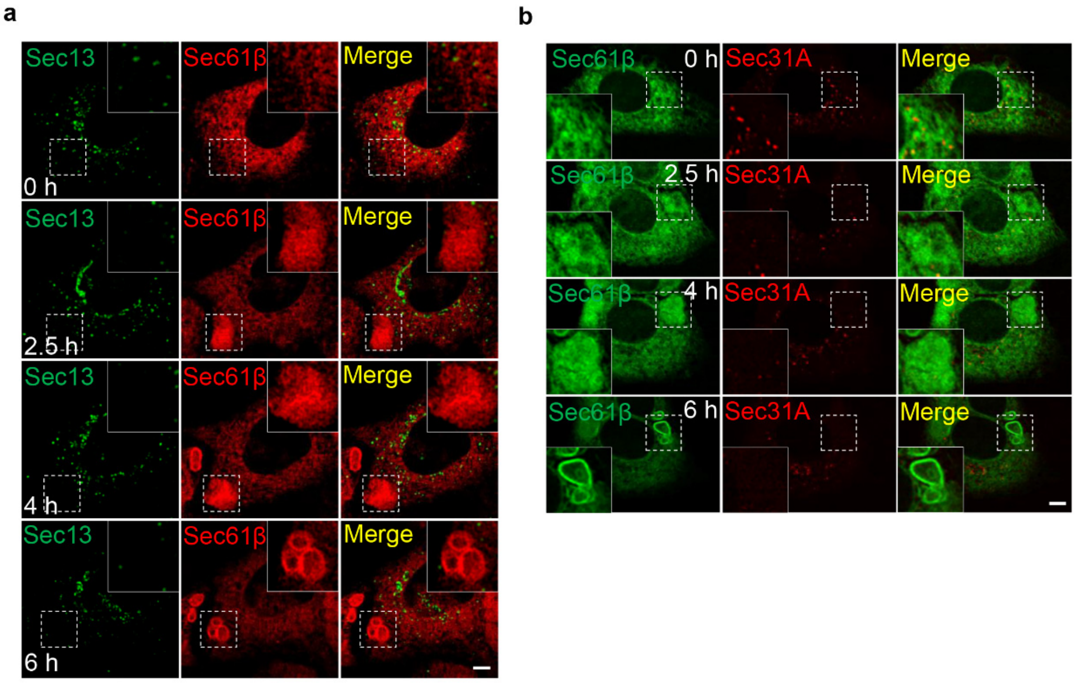

Supplementary information, Fig. S4 a NRK cells stably expressing Sec13-GFP and RFP-Sec61β were treated with Tg, and time-lapse images of a typical cell were acquired by Opera Phenix microscopy with 60× confocal mode. Scale bar, 5 μm. **b** GFP-Sec61β-expressing NRK cells transfected with mRuby-Sec31A were treated with Tg, and time-lapse images of a typical cell were acquired by Opera Phenix microscopy with 60× confocal mode. Scale bar, 5 μm.
