# Supplementary material for: COPII mitigates ER stress by promoting formation of ER whorls
Source: Cell Res. 2020 Sep 28;31(2):141–56. doi: 10.1038/s41422-020-00416-2 (PMC8026990; doi:10.1038/s41422-020-00416-2)
Supplement: Supplementary file 5 — Supplementary information, Figure S5 [file 41422_2020_416_MOESM5_ESM.pdf]

a

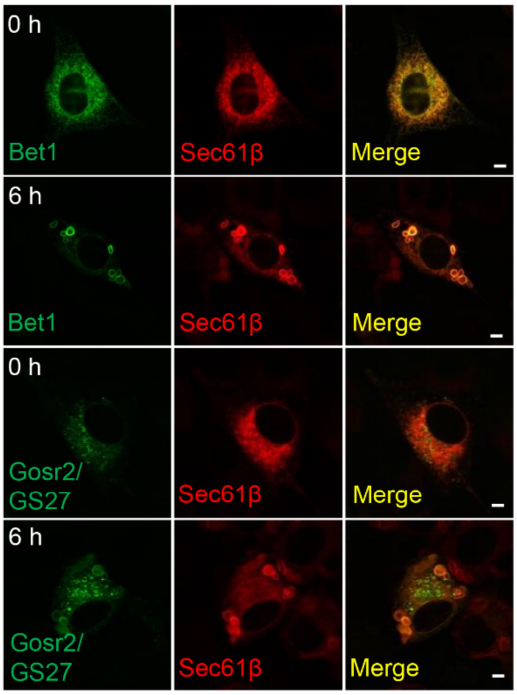

b

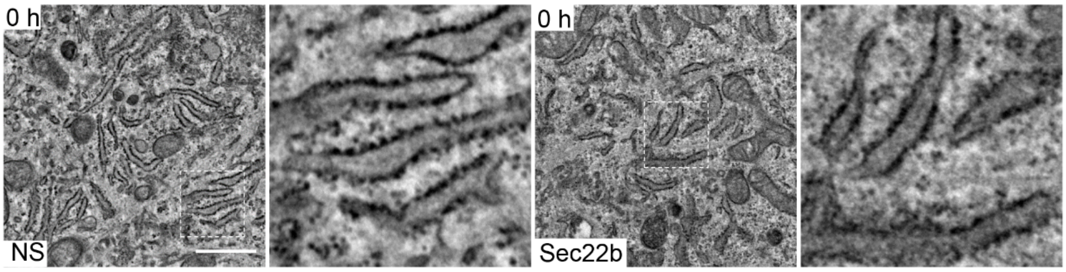

Supplementary information, Fig. S5 a RFP-Sec61β-expressing NRK cells transfected with GFP-Bet1 or GFP-Gosr2/GS27 were treated with Tg for 0 or 6 h and then observed by confocal microscopy. Scale bar, 5 µm. **b** RFP-Sec61β-expressing NRK cells were transfected with nonspecific (NS) or Sec22b RNAi, treated with Tg for 0 h, and then visualized by TEM. Scale bar, 1 µm.
