# Supplementary material for: COPII mitigates ER stress by promoting formation of ER whorls
Source: Cell Res. 2020 Sep 28;31(2):141–56. doi: 10.1038/s41422-020-00416-2 (PMC8026990; doi:10.1038/s41422-020-00416-2)
Supplement: Supplementary file 6 — Supplementary information, Figure S6 [file 41422_2020_416_MOESM6_ESM.pdf]

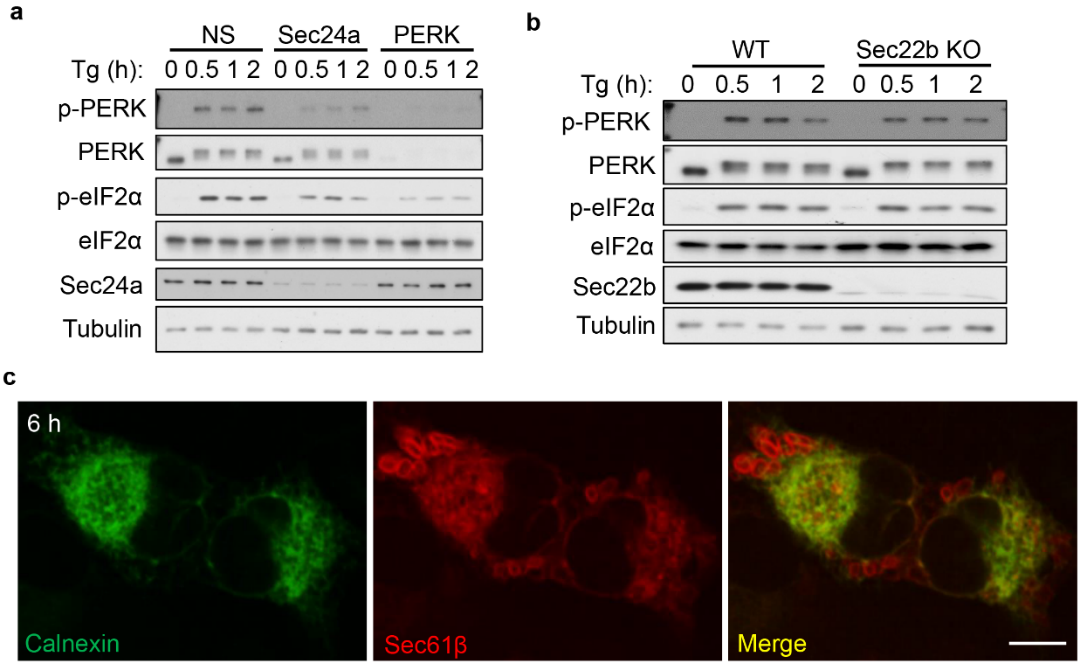

Supplementary information, Fig. S6 a NRK cells were transfected with nonspecific (NS), Sec24a, or PERK RNAi. Cells were treated with Tg for the indicated times and analyzed by western blot using an antibody against phospho-PERK (Thr980) or phospho-eIF2α (Ser51). **b** Wild-type and Sec22b KO NRK cells were treated with Tg for the indicated times and analyzed by western blot using an antibody against phospho-PERK (Thr980) or phospho-eIF2α (Ser51). **c** RFP-Sec61β-expressing NRK cells transfected with GFP-Calnexin were treated with Tg for 6 h and then observed by confocal microscopy. Scale bar, 10 µm.
