# Supplementary material for: COPII mitigates ER stress by promoting formation of ER whorls
Source: Cell Res. 2020 Sep 28;31(2):141–56. doi: 10.1038/s41422-020-00416-2 (PMC8026990; doi:10.1038/s41422-020-00416-2)
Supplement: Supplementary file 7 — Supplementary information, Figure S7 [file 41422_2020_416_MOESM7_ESM.pdf]

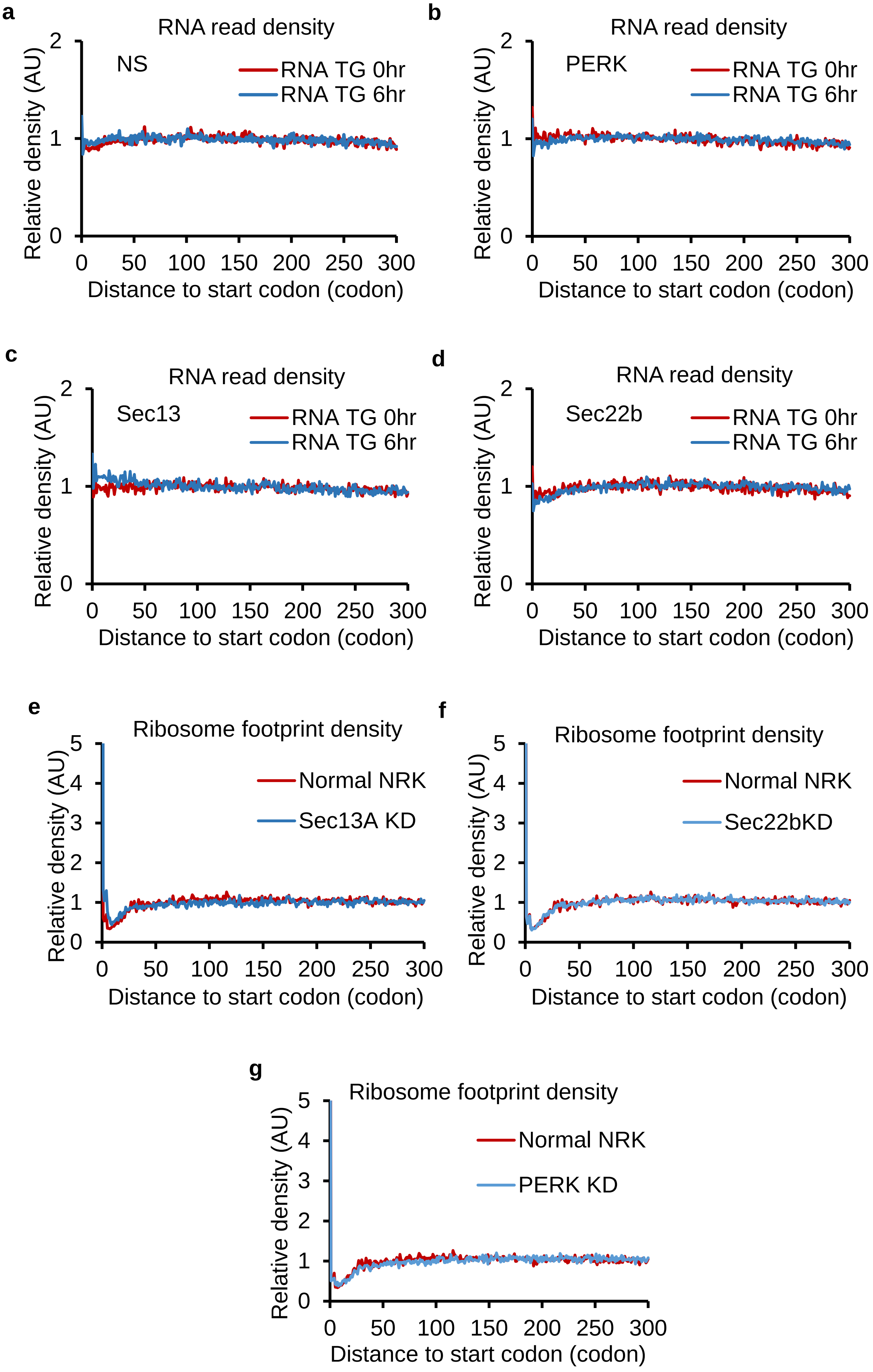

a
RNA read density
NS
RNA TG 0hr
RNA TG 6hr
Relative density (AU)
Distance to start codon (codon)
b
RNA read density
PERK
RNA TG 0hr
RNA TG 6hr
Relative density (AU)
Distance to start codon (codon)
c
RNA read density
Sec13
RNA TG 0hr
RNA TG 6hr
Relative density (AU)
Distance to start codon (codon)
d
RNA read density
Sec22b
RNA TG 0hr
RNA TG 6hr
Relative density (AU)
Distance to start codon (codon)
e
Ribosome footprint density
Normal NRK
Sec13A KD
Relative density (AU)
Distance to start codon (codon)
f
Ribosome footprint density
Normal NRK
Sec22bKD
Relative density (AU)
Distance to start codon (codon)
g
Ribosome footprint density
Normal NRK
PERK KD
Relative density (AU)
Distance to start codon (codon)

Supplementary information, Fig. S7 a-d Average density of RNA-seq reads on all coding genes aligned by their start codons for NRK cells treated with nonspecific siRNA (**a**), PERK siRNA (**b**), Sec13 siRNA (**c**), or Sec22b siRNA (**d**), before (0 h) and after (6 h) Tg treatment. For direct comparison with the RPF read densities shown in Fig. 7h-k, the RNA-seq reads were allocated to each codon based on their 5' end locations. The count of RNA-seq reads on each codon was normalized by the average count per codon after the first 30 codons. **e-g** Average density of ribosome footprints on all coding genes aligned by their start codons for normal NRK cells compared to cells with Sec13A knockdown (**e**), Sec22b knockdown (**f**), or PERK knockdown (**g**). The ribosome footprints were allocated to each codon according to their P-sites, and the count of footprints on each codon was normalized by the average count per codon after the first 30 codons.
