# Supplementary material for: COPII mitigates ER stress by promoting formation of ER whorls
Source: Cell Res. 2020 Sep 28;31(2):141–56. doi: 10.1038/s41422-020-00416-2 (PMC8026990; doi:10.1038/s41422-020-00416-2)
Supplement: Supplementary file 9 — Supplementary movie legend [file 41422_2020_416_MOESM9_ESM.pdf]

Supplementary information, Movie S1 The formation of ER whorls. NRK cells stably expressing GFP-Sec61β were treated with Tg and time-lapse images were acquired by Opera Phenix microscopy with 60× confocal mode. Scale bar, 5 μm.
